# ARHGEF2/EDN1 pathway participates in ER stress-related drug resistance of hepatocellular carcinoma by promoting angiogenesis and malignant proliferation

**DOI:** 10.1038/s41419-022-05099-8

**Published:** 2022-07-27

**Authors:** Yue Zhu, Weiwei Liu, Zishu Wang, Yanfei Wang, Chaisheng Tan, Zhipeng Pan, Anqi Wang, Jiatao Liu, Guoping Sun

**Affiliations:** 1 Department of Oncology, The First Affiliated Hospital of Anhui Medical University, Hefei, Anhui China; 2 Department of Otorhinolaryngology, Head and Neck Surgery, The First Affiliated Hospital of Anhui Medical University, Hefei, Anhui China; 3 Department of Oncology, The First Affiliated Hospital of Bengbu Medical College, Bengbu, Anhui China; 4 Department of Integrated Traditional Chinese and Western Medicine, Anhui Medical University, Hefei, Anhui China; 5 Department of Pharmacy, The First Affiliated Hospital of Anhui Medical University, Hefei, Anhui China

**Keywords:** Liver cancer, Oncogenes, Tumour angiogenesis

## Abstract

Endoplasmic reticulum (ER) stress is widely involved in the drug resistance of hepatocellular carcinoma (HCC), but the mechanism of ER stress-induced drug resistance involves multiple signaling pathways that cannot be fully explained. Exploring genes associated with ER stress could yield a novel therapeutic target for ER stress-induced drug resistance. By analyzing RNA-sequencing, ATAC-sequencing, and Chip-sequencing data of Tunicamycin (TM)-treated or untreated HCC cells, we found that Rho guanine nucleotide exchange factor 2 (ARHGEF2) is upregulated in HCC cells with ER stress. ARHGEF2 plays an active role in tumor malignant progression. Notwithstanding, no research has been done on the link between ER stress and ARHGEF2. The function of ARHGEF2 as a novel downstream effector of ER stress in the angiogenesis and treatment resistance of HCC was revealed in this work. ARHGEF2 overexpression was linked to malignant development and a poor prognosis in HCC. ER stress stimulates the expression of ARHGEF2 through upregulation of ZNF263. Elevated ARHGEF2 accelerates HCC angiogenesis via the EDN1 pathway, enhances HCC cell proliferation and tumor growth both in vitro and in vivo, and contributes to ER stress-related treatment resistance. HCC cell growth was more inhibited when ARHGEF2 knockdown was paired with targeted medicines. Collectively, we uncovered a previously hidden mechanism where ARHGEF2/EDN1 pathway promotes angiogenesis and participates in ER stress-related drug resistance in HCC.

## Introduction

Hepatocellular carcinoma (HCC) is one of the most prevailing malignant tumors in the world [1]. Despite the continued development of targeted and immunotherapeutic drugs in recent years, HCC has the third-highest death rate, at 8.3 percent, according to the global cancer statistics of 2020 [2]. Molecularly targeted agents, such as Lenvatinib, occupy the first-line position in the treatment of advanced, unresectable HCC, representing a significant breakthrough in the therapy of HCC. Despite the fact that although the median overall survival of Lenvatinib has been increased to 13.6 months [3], its long-term survival still does not satisfy the majority of patients, and there is drug resistance to the targeted therapy exists [4–6].

The link between HCC and endoplasmic reticulum (ER) stress is the focus of our research group. In response to ER stress, the unfolded protein response (UPR) is activated and can protect tumor cells by repairing damaged proteins or degrading misfolded proteins, as well as promote tumor invasion and metastasis in multiple ways [7–9]. Angiogenesis is the essential route by which ER stress promotes tumor progression. Angiogenesis is necessary for tumor survival, which leads to hematogenous metastasis and drug tolerance [10]. Angiogenesis boosted survival and decreased apoptosis of tumor cells, not only owing to the delivery of oxygen and nutrients but also relying on the paracrine release of anti-apoptotic substances from the endothelial cells in these new vessels [11]. Hence, inhibition of tumor cell proliferation and apoptosis resistance via the angiogenesis pathway can be considered [12]. Despite the fact that previous studies have demonstrated that ER stress contributes to angiogenesis in a variety of cancers [8, 13, 14], the mechanism by which ER stress induces angiogenesis leads to apoptosis resistance cannot be fully explained at this time.

Notably, ARHGEF2 is a member of the RhoGEF family of guanine nucleotide exchange factors. ARHGEF2 has been proven to have specific RhoA exchange activity by increasing the guanosine triphosphate (GTP) -binding form of RhoA [15], thereby regulating the cytoskeleton, morphology, and promotion of cell proliferation [15–17]. Early studies identified ARHGEF2 as a RhoA-activating enzyme, despite the fact that ARHGEF2 has since been shown to play a crucial role in the invasion and metastasis of a variety of cancers [18–21]. However, the involvement of ARHGEF2 in tumor angiogenesis is uncertain up to now. The link between ARHGEF2 related pathways and ER stress in HCC remains unspecified.

In the current study, we analyzed the RNA-sequencing, ATAC-sequencing, and Chip-sequencing data and discovered that ER-stressed HCC cells had a significant increase in ARHGEF2. Further experiments showed that ARHGEF2 was highly expressed in liver cancer and was associated with malignant progression and poor prognosis. ER stress up-regulated ARHGEF2 via the transcription factor ZNF263. Elevated ARHGEF2 accelerates angiogenesis by modulating mRNA and the protein expression levels of EDN1, promoting HCC cell proliferation and tumor formation both in vitro and in vivo. More importantly, ARHGEF2 participates in ER stress-related targeted drugs resistance. We concluded that ARHGEF2 was a novel target for antiangiogenic therapy in HCC and that inhibition of ARHGEF2 could reverse apoptosis resistance associated with ER stress.

## Materials and methods

### Immunohistochemistry (IHC)

A tissue microarray of human liver cancer and adjacent tissue was obtained from the First Affiliated Hospital of Anhui Medical University. The experiment was approved by the Ethics Committee. All clinical specimens were obtained from patients with written informed consent. IHC analysis staining was performed as previously described [7]. The antibodies in this study are shown in the Supplementary Table 1. The staining intensity was divided into 0–3 points, and the cell-positive rate was divided into 0–9 points (e.g., zero represents a 0–10% positive rate, and nine represents a 90–100% positive rate). We identified that the final IHC score of cancer tissue or adjacent tissue was the product of the scores of these two indicators [22]. Furthermore, we identified that an IHC score ≥10 was high expression, and an IHC score < 10 was low expression based on the median expression.

### Cell culture

The HepG2, MHCC97H, Hep3B and Huh7 cell lines were purchased from the Chinese Academy of Sciences (Shanghai, China) and validated for authentication using the short tandem repeat (STR) method. The HEK293T cells were from the American Type Culture Collection (Manassas, VA, USA). The HUVECs were obtained from Bioogenetech (Shanghai, China). These cells were cultured in a basic nutrient solution consisting of high-sugar Dulbecco’s Modified Eagle Medium (DMEM) (or Roswell Park Memorial Institute [RPMI]−1640), fetal bovine serum (Gibco) (10%), and dual antibiotics (penicillin and streptomycin, 1%), and the cells were placed in an incubator at 37 °C and 5% CO_2_.

### Western blot analysis

A western blot analysis was performed [7]. The antibodies in this study are shown in the Supplementary Table 1.

### Quantitative real-time PCR (qRT-PCR)

Total RNA for the qRT-PCR was isolated using TRIzol (Invitrogen, USA). The complementary DNA (cDNA) was reverse transcribed using HiScript II Q RT SuperMix for qRT-PCR (Vazyme, Nanjing, China) according to the manufacturer’s instructions. The analysis was performed using the AceQ qPCR SYBR Green Master Mix (Vazyme, Nanjing, China). The primer sequences are shown in the Supplementary Table 2.

### siRNA and plasmid transfection

ZNF263, ARHGEF2, EDN1, RhoA, and the negative control small interfering RNA were synthesized by GenePharma Company (Shanghai, China). The ZNF263 overexpression plasmid was constructed by Genomeditech (Shanghai, China). Transfection was performed using Lipofectamine™ 2000 (Invitrogen, USA) in strict accordance with the manufacturer’s instructions. The siRNA target sequence is described in Supplementary Table 3.

### Lentivirus infection

The lentivirus expressing short hairpin RNA (shRNA) targeting ARHGEF2 was designed and synthesized by GenePharma (Shanghai, China). The lentivirus overexpressing ARHGEF2 was designed and synthesized by Genechem (Shanghai, China). The efficiency of the lentivirus infection was validated using western blot and qRT-PCR.

### Dual-luciferase reporter assay

The ARHGEF2 promoter reporter gene vector was constructed. The predicted potential ZNF263 binding sites of the ARHGEF2 promoter were mutated. The wild-type or mutated ARHGEF2 promoter reporter vectors, pBabe or the pBabe-ZNF263 plasmid, were co-transfected into the 293 T cells and ER-stressed HepG2 cells. The dual-luciferase reporter gene assay system (Promega) was used to detect the luciferase activity.

### Conditioned medium (CM)

The HepG2 and MHCC97H cells were cultured to a 70–80% fusion and then cultured in serum-free DMEM for 24 h. The ARHGEF2 knockdown or overexpression cells were incubated under the same conditions. The culture medium was collected, centrifuged at 3000 rpm at 4 °C for 10 min, and the supernatant was retained, filtered, and stored at −80 °C.

### Chicken chorioallantoic membrane (CAM) assay

The chick embryo CAM model was established at the eighth day of age. A sterile gelatin sponge 1 mm^3^ in size soaked in prepared cell-conditioned medium was inoculated into the allantoic cavity of the specific pathogen-free (SPF) chicken embryos. After incubating them for seven days, the CAMs were cut out and harvested. The number of new blood vessels was counted.

### HUVEC tube formation assay

A total of 100 µL of Matrigel (Corning, USA) was added to a 48-well plate and incubated at 37 °C for 60 min. The HUVECs (1 × 10^4^) were seeded into wells with 300 µL prepared CM. After incubation at 37 °C for six hours, the capillary-like structures were scanned using a light microscope (Olympus), and the number of branches was analyzed using WimTube (https://www.wimasis.com).

### HUVEC transwell assay

The HUVECs were resuspended in a serum-free medium and placed in the upper chambers (3×10^4^ cells/well). The bottom chamber was filled with a medium with 10% FBS and conditioned medium (CM). After 24 h, the invasive cells were stained with crystal violet and counted. The invasion assay was the same as above, and the upper chamber was covered with 1:5 diluted Matrigel (Corning, USA).

### HUVEC wound-healing assay

The HUVECs cells were seeded into six-well plates (5 × 10^5^ cells/well), and serum-free medium was added for overnight serum starvation. The incubated cells were left to grow to 80% in an incubator at 37 °C. The wound was scratched using a 10 ul pipette tip and then cultured with CM for 24 h. Microscope images were taken at 0 h and 24 h after the scratch creation.

### Cell viability analysis

After stable transfection, the cells were seeded into 96-well plates (3 × 10^3^ cells/well). The cell proliferation rate was detected using CCK-8 solution (BestBio, Shanghai, China) after different treatments, and measure the absorbance at 450 nm.

### Colony formation assay

The stably transfected cells were seeded into six-well plates (5 × 10^2^ cells/well) and routinely cultured for 14 days, during which the fresh medium was changed until the cells formed stable colonies. After fixation with paraformaldehyde, crystal violet was used as a stain, and the cells were observed under a microscope (Olympus).

### Annexin V-FITC/PI apoptosis detection

Cells were collected in a reaction tube and prepared into a single cell suspension. After adding 5 μl of annexin V-FITC and PI (BD Biosciences, USA), light was avoided for 15 minutes. Apoptosis was detected using flow cytometry (Beckman Coulter, USA) and analyzed using CytoExpert.

### Immunofluorescence assay

Cells were fixed with 4% paraformaldehyde for 15 min. Goat serum was added on the slides for blocking for 30 min. The cells were then incubated with anti-ARHGEF2 primary antibodies overnight at 4 °C, followed by incubating with a corresponding secondary antibody. Subsequently, the cells were reacted with DAPI in the dark for 5 min. The images were observed under a laser confocal microscope (Leica).

### Animal experiment

BALB/c nude mice (4-5 weeks old, *n* = 42) were purchased from GemPharmatech Co., Ltd. (Nanjing, China). The mice were randomly assigned to groups. HepG2 cells (2 × 10^6^ cells) from the control group (shNC) and ARHGEF2 stable-knockdown group (shARHGEF2) were resuspended in 0.1 ml serum-free medium and subcutaneously injected into the right axilla of the mice. The same was true for the MHCC97H cells in the control group (Vector) and the ARHGEF2 overexpressed group (ARHGEF2). The tumor volume was determined every five days. After 20 days, the mice were euthanized, and the tumors were excised and weighed. In the lenvatinib drug experiment, mice were given lenvatinib 10 mg/kg orally from the time the tumor grew to 100 m^3^ for 21 consecutive days. The mice were euthanized, and the tumors were weighed. Investigators were blinded to the group allocation when assessing the results. The animal experiment was approved by the Animal Ethics Committee of Anhui Medical University.

### Statistical analyses

Single comparisons were performed using unpaired or paired Student’s t-tests, and multiple comparisons were performed using a one-way ANOVA with a post hoc test. GraphPad Prism 8 software (GradPad Software, Inc.) was used to evaluate the statistical significance. A *p* value <0.05 was considered to be statistically significant. Statistical significance is represented by: *p < 0.05, **p < 0.01, and ***p < 0.001.

## Results

### ER stress upregulated the expression of ARHGEF2 in HCC cells

**Fig. 1:**
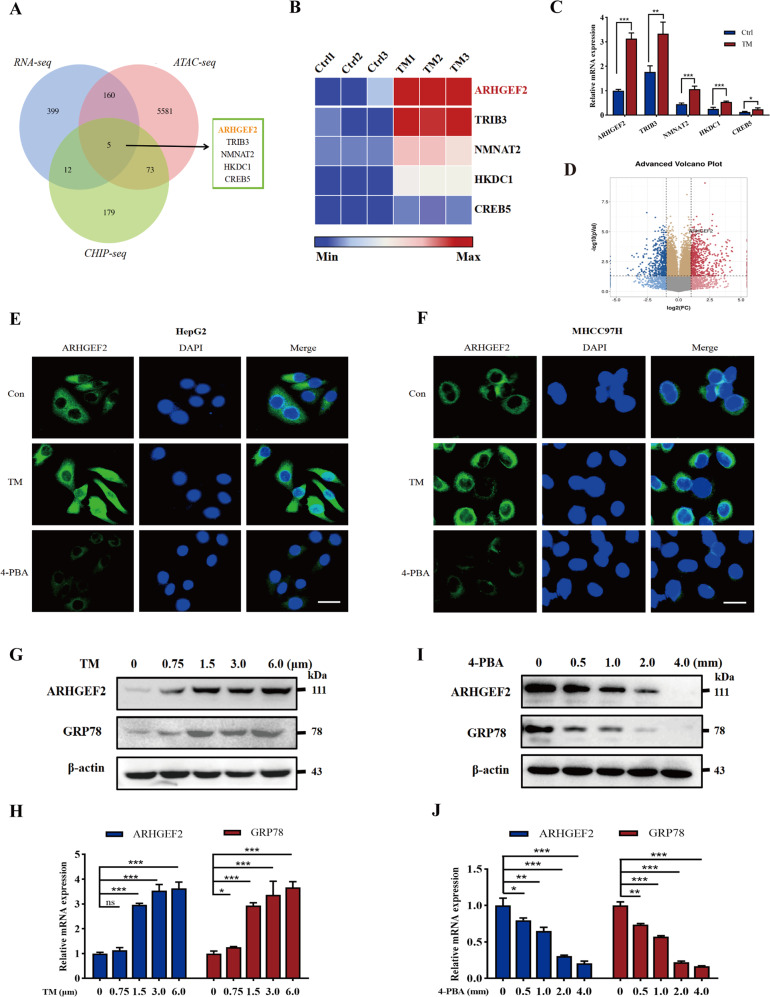
ER stress upregulated the expression of ARHGEF2 in HCC cells. **A** RNA-sequencing, ATAC-sequencing, and Chip-sequencing of TM-treated or untreated HepG2 cells were analyzed to find the differentially expressed genes. **B** A heat map was constructed according to the mRNA sequencing results. **C** qRT-PCR was used to detect the expression of differential genes in the TM-treated or untreated HepG2 cells. **D** Volcanic map of the ARHGEF2 expression. **E**, **F** HepG2 and MHCC97H cells were treated with TM or 4-PBA, and the expression of ARHGEF2 was detected by immunofluorescence. **G**, **H** Changes in ARHGEF2 and the GRP78 protein (**G**) and mRNA (**H**) level after the HepG2 cells were treated with different concentrations of TM. **I**, **J** After HepG2 was treated with 4-PBA at different concentrations, the protein (**I**) and the mRNA (**J**) level of ARHGEF2 and GRP78 were detected.

We analyzed the RNA-sequencing, ATAC-sequencing, and Chip-sequencing data of Tunicamycin (TM)-treated or untreated HepG2 cells to find the differentially expressed genes (Fig. 1A). Here, we list the five upregulated genes with TM treatment, including ARHGEF2, TRIB3, NMNAT2, HKDC1 and CREB5 (Fig. 1B). qRT-PCR analysis was performed to examine the expressions of these genes in response to TM in HepG2 cells (Fig. 1C). Then the upregulated gene, ARHGEF2, was screened (Fig. 1D). The immunofluorescence assay showed that the expression of ARHGEF2 was significantly increased after ER stress, whereas inhibition of ER stress with 4-Phenylbutyric acid (4-PBA) resulted in the downregulation of ARHGEF2 expression (Fig. 1E, F). In addition, both the western blot and qRT-PCR methods showed that the ARHGEF2 protein and the mRNA expression levels changed with an increasing TM concentration in HepG2 cells (Fig. 1G, H). With an increase in the 4-PBA concentration, the levels of the ARHGEF2 protein and mRNA decreased gradually (Fig. 1I, J). TCGA database also showed that ARHGEF2 was significantly correlated with ER stress marker proteins glucose-regulated protein 78 (GRP78), protein kinase RNA-like ER kinase (PERK), activating transcription factor 6 (ATF6), and inositol-requiring enzyme-1α (IRE1α), further proved that ARHGEF2 was regulated by ER stress (Supplementary Fig. 1A).

### ER stress upregulated ARHGEF2 expression through the activation of ZNF263

**Fig. 2:**
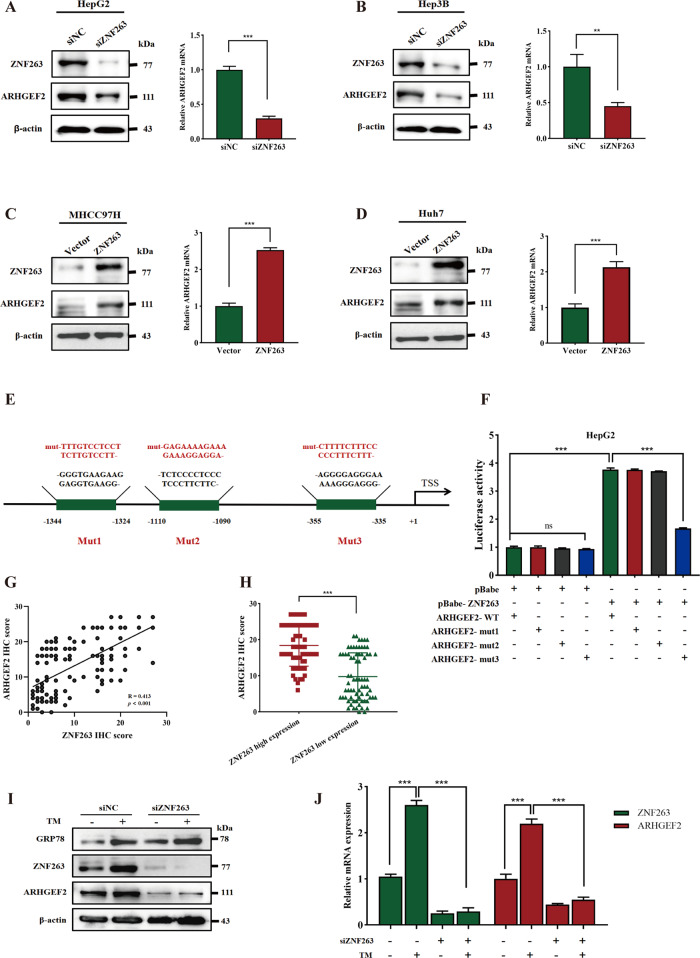
ER stress upregulated ARHGEF2 expression through the activation of ZNF263. **A**, **B** After knockdown of ZNF263 in the HepG2 cells (**A**) and the Hep3B cells (**B**), the levels of the ARHGEF2 protein and mRNA were detected using western blot and qRT-PCR. **C**, **D** After overexpression of ZNF263 in the MHCC97H cells (**C**) and the Huh7 cells (**D**), the levels of the ARHGEF2 protein and mRNA were detected using western blot and qRT-PCR. **E** Schematic representation of the putative three mutated (mut) ZNF263-binding sites in the promoter of the human ARHGEF2 gene from the Jaspar transcription profile database (http://jaspar.genereg.net). **F** Cells were co-transfected with ARHGEF2-luc, ARHGEF2-mut-R1, ARHGEF2-mut-R2, or ARHGEF2-mut-R3 together with pBabe-ZNF263 or pBabe. Relative luciferase activity was detected 24 h after transfection. **G** IHC was performed on liver cancer and adjacent tissues of 138 patients, and the correlation between ARHGEF2 and ZNF263 was analyzed. **H** IHC staining index of ARHGEF2 in the ZNF263 high/low group. **I**, **J** Western blot (**I**) and qRT-PCR (**J**) was used to detect whether TM induced the changes of the ZNF263 and ARHGEF2 level after ZNF263 knocked down.

Our group has previously confirmed that ZNF263 was a downstream effector of ER stress [23]. By analyzing the TCGA dataset, we found that ZNF263 showed positively correlated with GRP78, PERK, ATF6 and IRE1α (Supplementary Fig. 1B), while the western blot assay also confirmed that ZNF263 protein levels are regulated by ER stress (Supplementary Fig. 1C). Furthermore, ZNF263 was significant related to ARHGEF2 in HCC according to mRNA levels in TCGA database (Supplementary Fig. 1D). After measuring the mRNA expression of ZNF263 in different HCC cell lines by qRT-PCR (Supplementary Fig. 1E), we utilized ZNF263 small interfering RNA to transfect HepG2 and Hep3B cells. The results showed that the depletion of ZNF263 downregulated the expression of the ARHGEF2 protein and mRNA levels (Fig. 2A, B). We consistently overexpressed ZNF263 in the MHCC97H and Huh7 cells, and found that ZNF263 overexpression upregulated the expression level of ARHGEF2 (Fig. 2C, D). To further confirm the regulatory role of ZNF263 on ARHGEF2, we identified three binding sites using the Jaspar database (http://jaspar.genereg.net) (Supplementary Fig. 1F) that were mutated to construct the ARHGEF2-MUT1, ARHGEF2-MUT2, and ARHGEF2-MUT3 reporter genes (Fig. 2E). Increased luciferase activity in 293 T cells and ER-stressed HepG2 cells indicated significant transcriptional activation of ARHGEF2. In addition, when the putative ZNF263 binding site, R3, was mutated, the transcriptional activity was significantly reduced, while neither mutant Mut1 nor Mut2 were altered. This result suggested that R3 ( − 355/−335, AGGGGAGGGAAAAAAGGGGGGG) may be the binding site for ZNF263 (Fig. 2F, Supplementary Fig. 1G). IHC was performed on the tissue microarrays of 138 patients with HCC. ARHGEF2 showed a tendency of higher expression in the ZNF263 high expression group, with a positive correlation between them (Fig. 2G, H). In addition, western blot showed that after interfering with ZNF263 in the HepG2 cells, treatment with TM induced ER stress could barely cause the upregulation of the expression of ARHGEF2 (Fig. 2I, J).

### ARHGEF2 was highly expressed in HCC and was associated with a poor prognosis

**Fig. 3:**
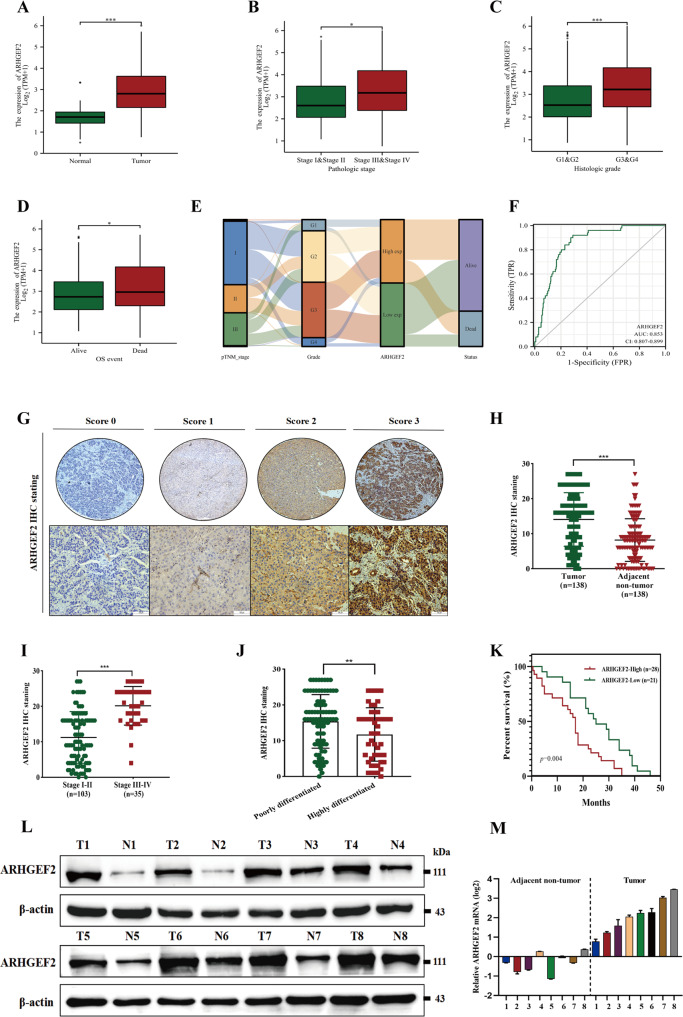
ARHGEF2 was highly expressed in HCC and was associated with poor prognosis. **A** Expression profile of ARHGEF2 mRNA in HCC tissues and normal tissues (TCGA). **B** ARHGEF2 expression in the different clinical stages (TCGA). **C** ARHGEF2 expression in different histological grades gruops (TCGA). **D** ARHGEF2 expression in different clinical status (TCGA). **E** Sankey diagram were made according to TNM stage, histological grade, status and ARHGEF2 expression from the TCGA dataset (https://portal.gdc.com). Each row represents a feature variable, different color represents different typing or stage, lines repersent the distribution of the same sample in different feature variables. **F** ROC curve was constructed based on the TCGA databases. **G** IHC staining were divided into four grades according to the staining intensity: 0, 1, 2 and 3. **H** IHC staining of ARHGEF2 in liver cancer and adjacent tissue. **I**, **J** IHC staining of ARHGEF2 in the different clinical stages (**I**) and histological grade (**J**). **K** Kaplan-Meier analysis of overall survival stratified by low ARHGEF2 expression (*n* = 21) and high ARHGEF2 expression (*n* = 28). **L** ARHGEF2 protein expression in 8 paired liver cancer and adjacent tissues was detected by Western Blot. **M** qRT-PCR was used to detect the mRNA expression of ARHGEF2 in liver cancer and adjacent tissues.

According to the results from the TCGA database, ARHGEF2 was highly expressed in a variety of different tumor tissues (Supplementary Fig. 2A). Moreover, the analysis of publicly available datasets TCGA (Fig. 3A), GSE36376, GSE10143 and GSE45267 (Supplementary Fig. 2B–D) showed that ARHGEF2 was remarkably increased in HCC tissues compared with normal tissues. The expression of ARHGEF2 correlated with the clinical stage (Fig. 3B), histological grade (Fig. 3C), and the clinical status (Fig. 3D, E). Furthermore, ROC curve was constructed based on the TCGA databases, suggested that ARHGEF2 has good sensitivity and specificity as diagnostic marker for HCC (Fig. 3F). Immunohistochemical staining was performed on the microarrays of 138 patients with HCC and adjacent tissues, and these were divided into four grades according to the staining intensity: 0, 1, 2, and 3 (Fig. 3G). IHC results confirmed that ARHGEF2 was overexpressed in HCC (Fig. 3H) and correlated with clinical stage (Fig. 3I) and histological grade (Fig. 3J). The Kaplan-Meier survival curve analysis illustrated that high ARHGEF2 level served as a factor for poor prognosis (Fig. 3K). In addition, our group collected eight pairs of fresh liver cancer tissues and adjacent tissues for the western blot and qRT-PCR detection. The results also showed that the expression of ARHGEF2 in the HCC tissues was significantly higher than that in the adjacent tissues (Fig. 3L, M).

### ARHGEF2 promoted proliferation of HCC in vivo and in vitro

**Fig. 4:**
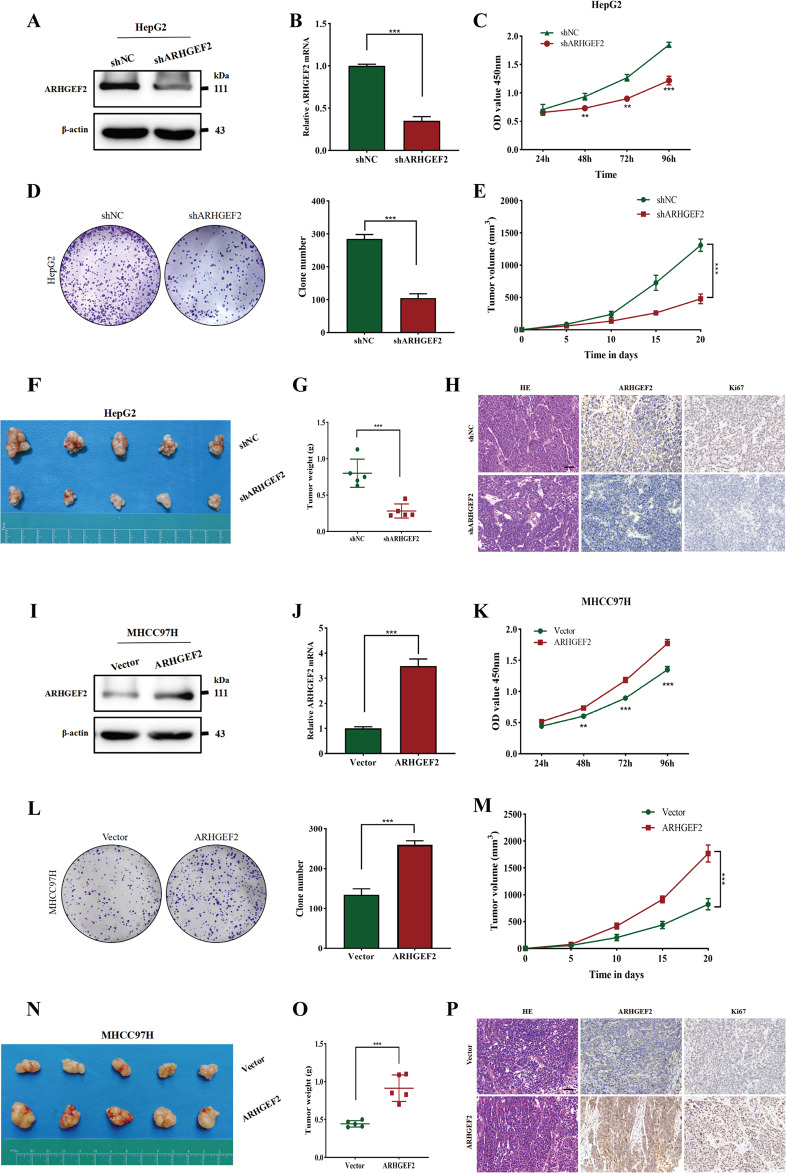
ARHGEF2 promoted proliferation of HCC in vivo and in vitro. **A**, **B** ARHGEF2 expression levels were confirmed by Western Blot (**A**) and qRT-PCR (**B**) after ARHGEF2 knockdown in the HepG2 cell line. **C**, **D** The proliferation of shNC and shARHGEF2 groups of the HepG2 cells was detected by CCK-8 (**C**) and clone formation assay (**D**). **E**–**G** Tumors from mice in shNC and shARHGEF2 groups (**F**), tumor volumes (**E**) and weights (**G**) were measured. **H** HE staining and IHC staining of ARHGEF2 and Ki67 indices were performed on the tumors of shNC and shARHGEF2 groups. **I**, **J** ARHGEF2 expression levels were detected by Western Blot (**I**) and qRT-PCR (**J**) after ARHGEF2 overexpression in the MHCC97H cell line. **K**, **L** CCK-8 (**K**) and clone formation assay (**L**) were used to detect the proliferation of Vector and ARHGEF2 groups of MHCC97H cells. **M**–**O** Tumors from mice in Vector and ARHGEF2 groups (**N**), tumor volumes (**M**) and weights (**O**) were measured. **P** HE staining and IHC staining on the tumors of Vector and ARHGEF2 groups.

To explore the effect of ARHGEF2 on the proliferation of HCC cells, western blot and qRT-PCR were used to detect the expression of ARHGEF2 in different HCC cell lines (Supplementary Fig. 2E, F). We then utilized the lentiviral technique to interfere with ARHGEF2 expression in the HepG2 cell line. Down-regulated expression of ARHGEF2 was confirmed by western blot (Fig. 4A), qRT-PCR (Fig. 4B), and immunofluorescence (Supplementary Fig. 2G). The CCK-8 assay and clone formation assay showed that interference with ARHGEF2 significantly inhibited the proliferation of HepG2 cells in vitro (Fig. 4C, D). Animal experiments further showed that ARHGEF2 knockdown inhibited HepG2 tumorigenesis in vivo (Fig. 4E–G). The IHC of transplanted tumors in mice showed that the expression of Ki67 was lower in the ARHGEF2 knockdown group than that in the control group (Fig. 4H). Futhermore, we overexpressd ARHGEF2 in the MHCC97H cell line (Fig. 4I–J, Supplementary Fig. 2H). The CCK-8 assay and clone formation assay showed that overexpression of ARHGEF2 enhanced the proliferation of MHCC97H cells (Fig. 4K, L). Animal experiments further demonstrated that the tumor sizes and weights of the ARHGEF2 group were obviously greater than those in the control group (Fig. 4M–O). The IHC of transplanted tumors showed that the expression of Ki67 was higher in the ARHGEF2 overexpressed group than that in the control group (Fig. 4P).

### ARHGEF2 as a new target for promoting angiogenesis

**Fig. 5:**
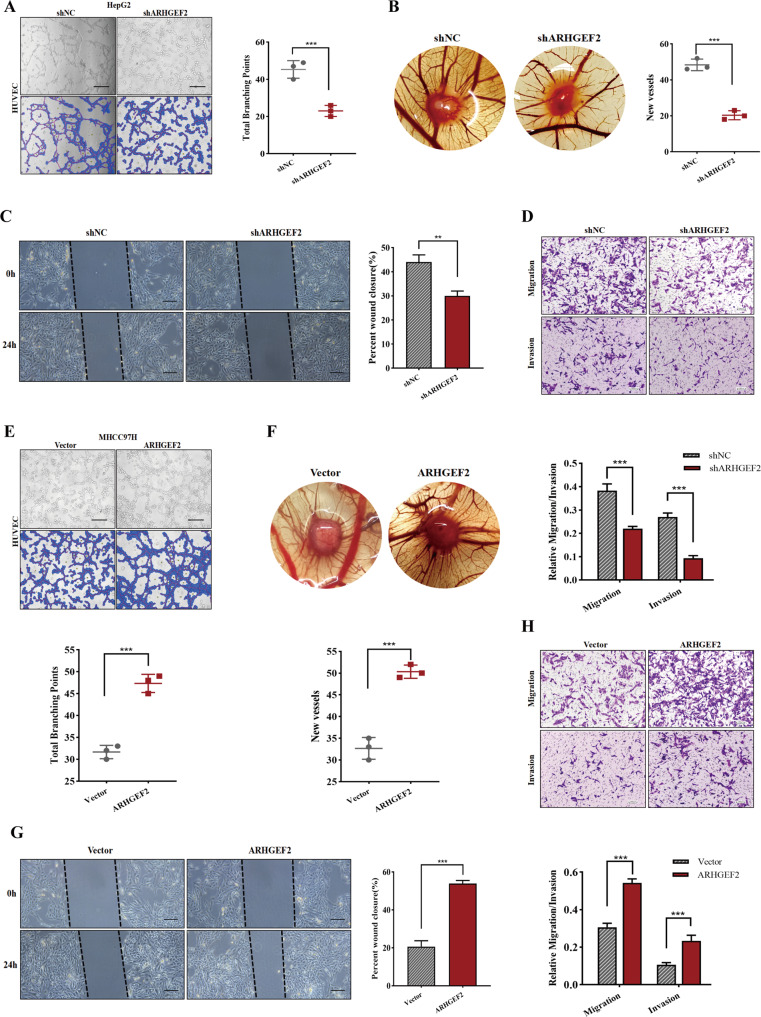
ARHGEF2 as a new target for promoting angiogenesis. **A** The cell-conditioned media of HepG2/shNC and HepG2/shARHGEF2 cells were collected for tube formation assay. **B** Representative images of CAM blood vessels stimulated with cell-conditioned media from the indicated cells. **C**, **D** The migration and invasion ability of CM-treated HUVEC cells were detected by wound-healing assay and transwell assay. **E**–**H** The cell-conditioned media of MHCC97H/Vector and MHCC97H/ARHGEF2 cells were collected for tube formation assay (**E**), CAM assay (**F**), wound-healing assay (**G**) and transwell assay (**H**).

The gene set enrichment analysis (GESA) suggested that ARHGEF2 may be involved in angiogenesis (Supplementary Fig. 2I–J). To further investigate the role of ARHGEF2 in the angiogenesis of HCC, we collected cell conditioned medium (CM) for an HUVEC tube formation assay. As shown in Fig. 5A, the HUVEC tube formation was decreased by adding the prepared ARHGEF2-knockdown HepG2 cells supernatants, and this led to much fewer total branching points as quantified by the WimTube (https://www.wimasis.com/en/WimTube). Furthermore, we examined the effect of ARHGEF2 on angiogenesis in vivo and evaluated new vessel formation. The CAM experiments suggested that the supernatant of the ARHGEF2-knockdown cells induced a lower angiogenic response than in the control cells (Fig. 5B). Moreover, the wound-healing assay (Fig. 5C) and the transwell assay (Fig. 5D) of the HUVECs further showed that ARHGEF2 knockdown inhibited the migration and invasion of HUVEC cells. Conversely, the MHCC97H supernatant with ARHGEF2 overexpression increased tube formation (Fig. 5E), neovascularization (Fig. 5F) and promoted the migration and invasion of HUVEC cells (Fig. 5G, H).

### ARHGEF2 was involved in the resistance of lenvatinib

**Fig. 6:**
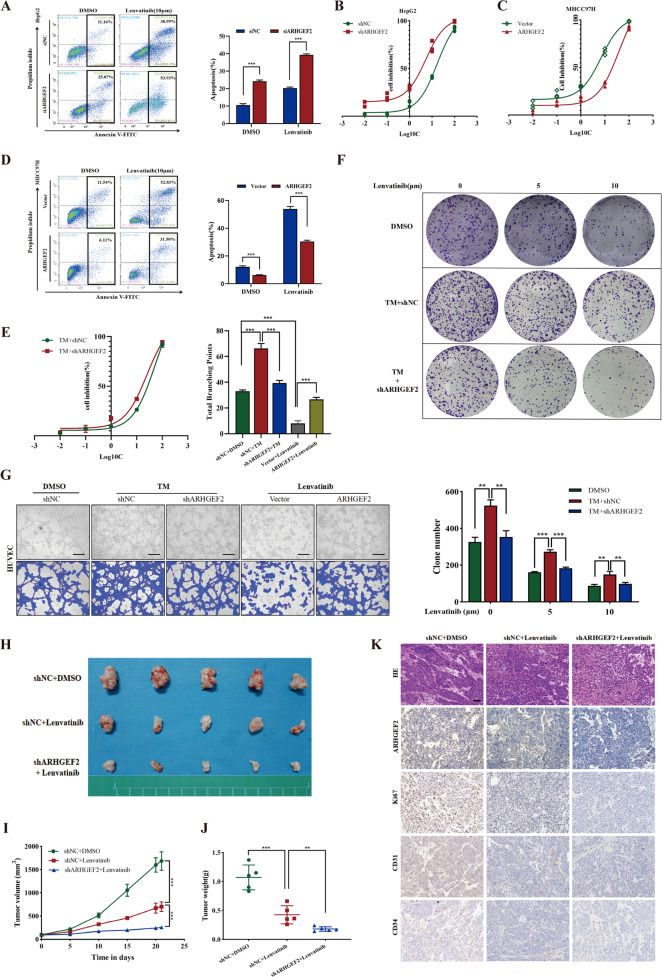
ARHGEF2 was involved in the resistance of lenvatinib. **A** After interfering with the expression of ARHGEF2 in the HepG2 cell line, detected the sensitivity of cells to lenvatinib by flow cytometry. **B** The IC50 value of lenvatinib was measured in ARHGEF2 knockdown and control HepG2 cells. **C** The IC50 value of lenvatinib in ARHGEF2 overexpression and control MHCC97H cells. **D** Flow cytometry were used to detected the sensitivity of cells to lenvatinib in ARHGEF2 overexpression and control MHCC97H cells. **E** The IC50 value of lenvatinib in TM-treated HepG2 cells with or without ARHGEF2 knockdown. **F** The proliferation ability of the HepG2 cells with different treatments were detected by clone formation assay. **G** Representative images of tube forming ability of HUVEC cells treated with different CM. **H** Tumors from mice after different treatments. **I**, **J** Measurement of tumor volume (**I**) and weight (**J**). **K** The expressions of Ki67, CD31, CD34 and ARHGEF2 in different groups were detected by IHC.

To explore the clinical value of ARHGEF2 in promoting cell proliferation and angiogenesis, we further investigated whether ARHGEF2 was associated with resistance to lenvatinib. As shown in Supplementary Fig. 3A, the IC50 of lenvatinib was found to be lower in the MHCC97H cell line with a low expression of ARHGEF2 compared to the high-expression cell line HepG2. After interfering with the expression of ARHGEF2 in the HepG2 cell line, we detected the cell apoptosis and found that the sensitivity of cells to lenvatinib increased (Fig. 6A). CCK-8 also proved that lenvatinib inhibited cell viability better after interfering with ARHGEF2 (Fig. 6B). Then, after the overexpression of ARHGEF2 in MHCC97H, the sensitivity of cells to lenvatinib decreased, and the effect of lenvatinib was significantly weakened (Fig. 6C, D). The clonal formation assay, CCK-8 and flow cytometry assay showed that TM-induced ER stress promoted cell proliferation and inhibited cell apoptosis, futher reduced the sensitivity of HCC cells to lenvatinib. However, this process was partially inhibited by interfering with ARHGEF2 (Fig. 6E–F, Supplementary Fig. 3B). As shown in Fig. 6G and Supplementary Fig. 3C, the ability of tube formation and neovascularization was enhanced by ER stress, and this was significantly inhibited by knockdown of ARHGEF2. Similarly, the overexpression of ARHGEF2 resisted the inhibitory effect of lenvatinib on tube formation (Fig. 6G), indicating that ER stress may promote angiogenesis through ARHGEF2 and further participate in resistance to lenvatinib. To further investigate the role of ARHGEF2 on the sensitivity of lenvatinib in vivo, we established xenograft models using BALB/c-nu mice. The result showed that, compared with the control group and the lenvatinib monotherapy group, the combination of the knockdown ARHGEF2 with lenvatinib achieved better tumor inhibition in vivo (Fig. 6H–J). The IHC demonstrated that the expression of Ki67, CD31 and CD34 were lower in the combined group than in the two others (Fig. 6K).

### EDN1 was the downstream effector of ARHGEF2

**Fig. 7:**
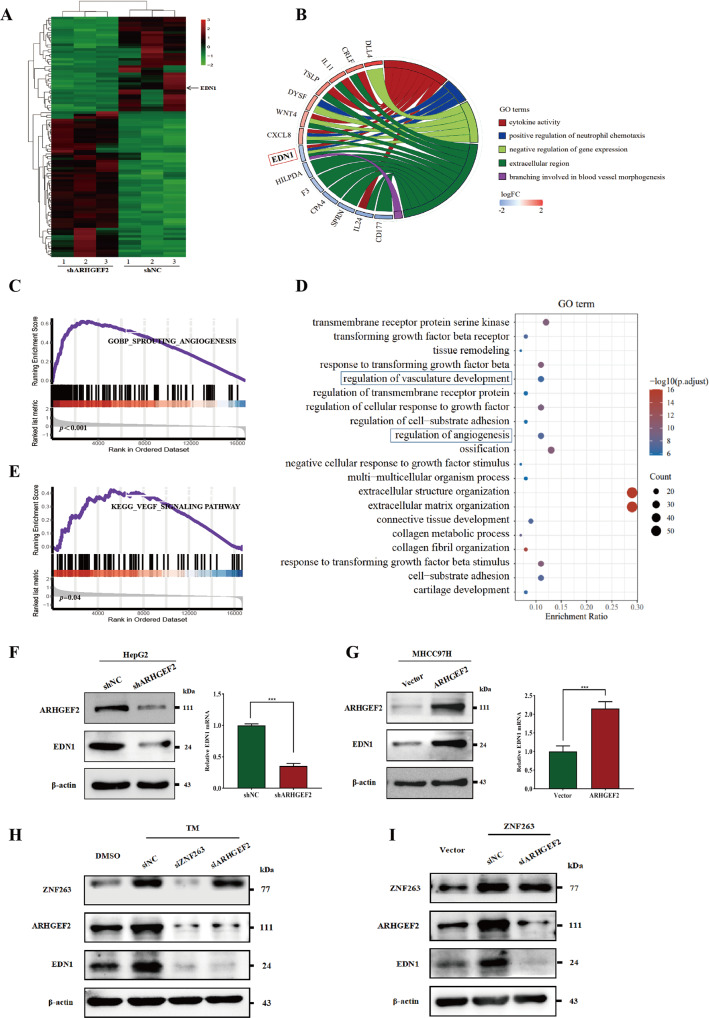
EDN1 was the downstream effector of ARHGEF2. **A** Heatmap of the differentially expressed genes after ARHGEF2 knocked down in the HepG2 cells. **B** The chord diagram was plotted by GO enrichment analysis. **C** GSEA plot, demonstrating a significant correlation between the EDN1 expression levels and sprouting angiogenesis from published datasets. **D** Go enrichment analysis revealed the EDN1-associated signaling pathways (TCGA). **E** A GESA plot regarding the correlation between EDN1 expression and the VEGF signaling pathway. **F** The effect of ARHGEF2 knockdown on EDN1 protein and mRNA level were examined by Western blot and qRT-PCR in HepG2 cell lines. **G** EDN1 protein and mRNA level after ARHGEF2 overexpression were detected in MHCC97H cell lines. **H** Western blot was used to detect EDN1 levels after ZNF263 and ARHGEF2 were knocked down under ER stress. **I** EDN1 levels were examined after ARHGEF2 knockdown in ZNF263 overexpressing cell lines.

To explore downstream target genes of the ARHGEF2 pathway, we first analyzed differences in gene expression profiles between ARHGEF2-knockdown cells and the control cells using RNA sequencing. Relative to the control cells, 272 DEGs (156 upregulated genes and 116 downregulated genes) were identified (Fig. 7A). We made a gene ontology (GO) chord diagram based on enriched pathways of ARHGEF2 and found that EDN1 is a common gene of multiple pathways (Fig. 7B). Thus, we considered EDN1 a promising potential effector of the ZNF263/ARHGEF2 signaling pathway. The GESA and GO enrichment indicated that EDN1 was involved in angiogenesis (Fig. 7C, D) and was closely related to the VEGF pathway (Fig. 7E). We detected the protein and mRNA expression of EDN1 in ARHGEF2 knockdown or overexpression HCC cells, showed that knockdown ARHGEF2 led to downregulation of EDN1 (Fig. 7F, Supplementary Fig. 4A), while overexpression with ARHGEF2 upregulated the protein and mRNA expression of EDN1 (Fig. 7G, Supplementary Fig. 4B). Western blot later exhibited that ER stress might increase the production of the EDN1 protein, but 4-PBA suppressed EDN1 protein levels (Supplementary Fig. 4C). We added TM to induced ER stress in ZNF263 or ARHGEF2 knockdown cell lines and worked out that the regulatory effect of ER stress on EDN1 was significantly inhibited, suggesting that ER stress regulates EDN1 via the ZNF263/ARHGEF2 pathway (Fig. 7H). Besides, to determine whether there is a direct transcriptional regulation of EDN1 by ZNF263, we knocked down ARHGEF2 in HepG2 cells overexpressing ZNF263. ARHGEF2 interference was found to be capable of reversing ZNF263’s upregulation of EDN1 (Fig. 7I). Furthermore, we also interfered with RhoA, a downstream gene of ARHGEF2, to verify whether the regulatory of ARHGEF2 on EDN1 was related to RhoA. The western blot and qRT-PCR showed that RhoA levels had no effect on EDN1 expression in HepG2 cell line (Supplementary Fig. 4D), suggested that ARHGEF2 regulates EDN1 was a RhoA-independent mechanism.

### ARHGEF2 exerted an angiogenic effect via EDN1

**Fig. 8:**
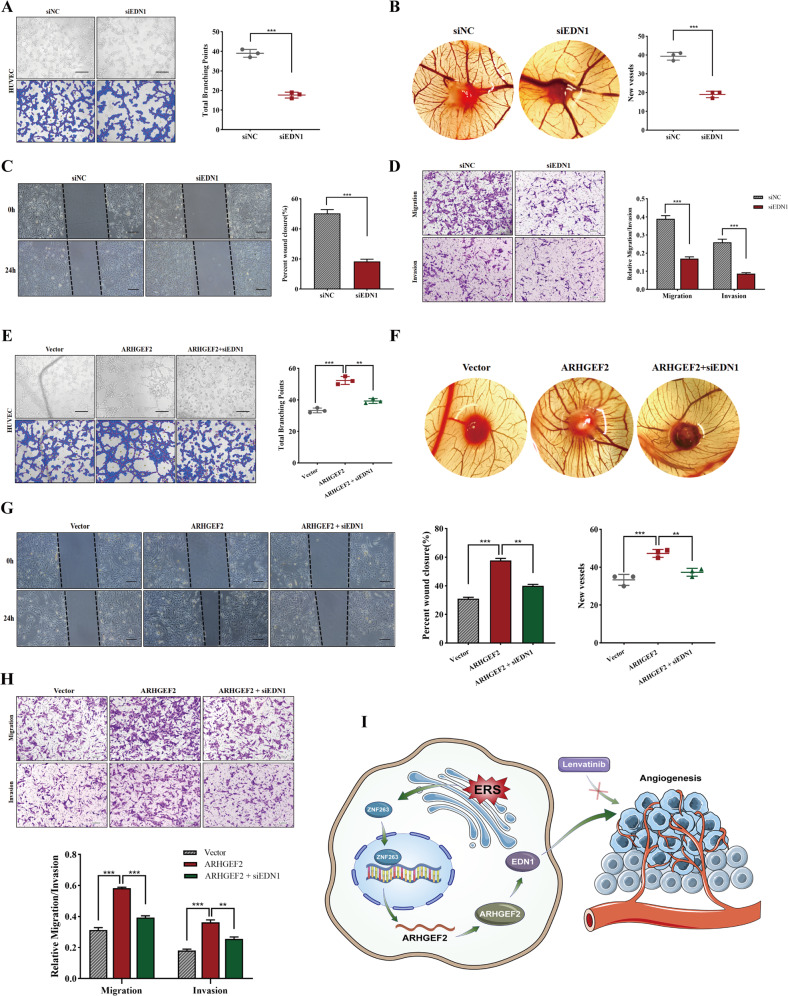
ARHGEF2 exerted an angiogenic effect via EDN1. **A**–**D** The cell-conditioned media of HepG2/siNC and HepG2/siEDN1 cells were collected for the tube formation assay (**A**), the CAM assay (**B**), the wound-healing (**C**) and the transwell assay (**D**). **E**–**H** EDN1 was knocked down in ARHGEF2 stable-overexpressing cell lines, and the CM was collected for the tube formation assay (**E**), the CAM assay (**F**), the wound-healing assay (**G**) and the transwell assay (**H**). **I** Schematic illustration of ER stress upregulates ZNF263/ARHGEF2/EDN1 signaling pathway to promotes angiogenesis and antagonizes the sensitivity of lenvatinib to HCC cells.

In order to further verify the role of EDN1 in promoting angiogenesis in HCC, we transfected EDN1 with small interfering RNA in HepG2 cell line, and collected conditioned medium for the tube formation assay, CAM assay, wound-healing assay and transwell assay. The results showed that EDN1 knocked down significantly inhibited the ability of tube formation (Fig. 8A). Similarly, the number of new blood vessels in chicken embryos (Fig. 8B) and the migration and invasion of HUVEC cells (Fig. 8C, D) were also suppressed. And then, we knocked down EDN1 after constructing cell lines stably overexpressing ARHGEF2. The HUVEC tube formation assay showed that ARHGEF2 overexpression significantly promoted microtubule formation compared with the control cells, but this function was inhibited by EDN1 interference (Fig. 8E). The CAM assay (Fig. 8F), wound healing (Fig. 8G) and transwell assay (Fig. 8H) suggested that the ability of ARHGEF2 to promotes HUVEC cell invasion, migration, and angiogenesis was inhibited to varying degrees after EDN1 knockdown.

## Discussion

In accordance with an increasing body of data, ER stress is implicated in tumor angiogenesis and treatment resistance [10, 23]. Although ER stress promotes tumor malignancy in a variety of ways, angiogenesis is still an important relationship to consider. Recent animal studies demonstrate that ER stress increases tumor vascular development under hypoxic settings [24], which is required for tumor growth and resistance to apoptosis [25]. However, the mechanism by which ER stress-induced angiogenesis leads to apoptosis resistance in HCC tumor cells is not fully understood.

By analyzing the sequencing results following ER stress in HCC cells and confirming differentially expressed genes, we were finally able to identify ARHGEF2. ARHGEF2 was found to be regulated by ER stress and overexpressed in HCC tissues, and its expression was correlated with a poor prognosis, clinical stage, and histological grades. Previous studies have substantiated that ARHGEF2 has unique RhoA exchange activity that facilitates the exchange of guanosine diphosphate (GDP) and GTP, thus promoting RhoA activation. As attested by the study, ARHGEF2 plays a key role in a variety of cancers by activating the RhoA signaling pathway, which may contribute to the proliferation and metastasis of cancer cells [18–21]. It is worth mentioning that ARHGEF2 was further studied as a RhoA-activating enzyme in already published literature. We proposed for the first time that ARHGEF2 is regulated by ER stress and specifically discussed its transcriptional regulation mechanism in HCC. Previous research in our group has shown that ZNF263 is overexpressed in liver cancer and promotes the occurrence and development of HCC [23]. Using Jaspar database, we predicted that ZNF263 might be an upstream transcription factor of ARHGEF2, and found the direct binding site of ZNF263 on the ARHGEF2 promoter using double luciferase, which further clarified the upstream regulatory signaling of ARHGEF2 in HCC.

Notably, our study also suggested for the first time that ARHGEF2 promotes liver cancer-related angiogenesis. We were able to create a stable HepG2 cell line with ARHGEF2 knockdown and an MHCC97H cell line with ARHGEF2 overexpression and found that ARHGEF2 could not only regulate the proliferation of HCC cells but also effectively stimulate the migration, invasion, and angiogenesis of HUVECs. Previous research has shown that RhoA was an important mediator in many angiogenesis processes, including endothelial cell migration, proliferation, as well as cell permeability [26–28]. It should be highlighted that the effect of ARHGEF2 is not in every respect dependent on RhoA activation, and its function involves RhoA-dependent and RhoA-independent mechanisms. For instance, ARHGEF2 performs a vitally important function in the affirmative feedback loop of the RAS/MAPK pathway that is independent of its RhoGEF activity [29]. As a downstream of ARHGEF2, RhoA has been extensively discussed and does not pique our interest. Interestingly, we knocked down ARHGEF2 for transcriptome sequencing, and found that the mRNA level of endothelin-1 (EDN1) were significantly inhibited, while RhoA could not effect the expression of EDN1, indicating that ARHGEF2 control of EDN1 may be a RhoA-independent mechanism.

EDN1 has been shown to be overexpressed in a variety of solid tumors [30–33]. Endothelins (EDNS) had three subtypes: EDN1, EDN2, and EDN3. EDN1 is the most prevalent subtype in the family [34] since it is the initial step in the creation of the peptide hormone Endothelin1 (ET-1), which promotes carcinogenesis and cancer development, particularly tumor angiogenesis [35, 36]. EDN1 has been further investigated as a target for anti-vascular therapy in cancers such as gastric cancer [37], breast cancer [38], and colon cancer [39]. Wang et al. found that EDN1 and VEGF, as leading angiogenic factors for angiogenesis, are supervised by miR-1 and promote angiogenesis hang around gastric cancer [37]. Except for that, it has been proven that melatonin inhibits the release of ET-1 in vitro by inhibiting the EDN-1 mRNA level, thus inhibiting the growth, angiogenesis, and metastasis of colon cancer [39]. These achievements put forward that EDN1 may be a potential target for anti-tumor growth and angiogenesis. Our study authenticated that the mRNA and protein levels of EDN1 were regulated by ARHGEF2. The ability of ARHGEF2 to promote HUVEC cell migration, invasion, and neovascularization were inhibited to varying degrees after EDN1 interference, indicating that the effect of ARHGEF2 on angiogenesis was EDN1-dependent.

As Molecular targeted drugs, such as Lenvatinib and Sorafenib were multi-target receptor tyrosine kinase inhibitors that act on multiple targets, including the vascular endothelial growth factor receptor, the fibroblast growth factor receptor, and the platelet-derived growth factor receptor [40], while its antitumor activity relies mainly on the inhibition of neovascularization to promotes cell apoptosis [41, 42]. Hence, we managed to explore the role of ARHGEF2 in promoting angiogenesis in targeted drug resistance. Our research completed proved that the overexpression of ARHGEF2 reduces the sensitivity of Lenvatinib in HCC cells. Inhibiting ARHGEF2 combined with Lenvatinib can significantly weaken the proliferation of HCC cells in vivo and in vitro. By inhibiting ARHGEF2, ER-stress-induced Lenvatinib resistance in tumor cells is reversed. It thus may be anticipated that ARHGEF2 specific inhibitors can overcome the defect of lenvatinib in the anti-tumor therapy of ER stress-related apoptosis resistance.

In the study, we brought to light that ARHGEF2 was regulated by ER stress and marked up the invasion and migration abilities of HUVEC cells to promote angiogenesis and tumor growth, further participating in the resistance of HCC cells to molecularly targeted drugs mediated by ER stress. Mechanistically, the transcription factor ZNF263 binds to the ARHGEF2 promoter and increases ARHGEF2 expression in response to ER stress. ARHGEF2 promotes angiogenesis through the EDN1 pathway, and the regulation of EDN1 was RhoA-independent. Despite the fact that our findings revealed a framework in which the ARHGEF2/EDN1 pathway contributes to ER stress-related drug resistance in HCC by promoting angiogenesis, several questions remain. We cannot rule out the possibility of additional mechanisms by which ARHGEF2 promotes angiogenesis, given its unique role in activating RhoA. The complicated relationship between ARHGEF2 and angiogenesis requires further investigation. Possibly multiple mechanisms together are activated to promote angiogenesis and HCC progression rather than being associated with the regulation of a single pathway. Notwithstanding, this study provided a new insight into the potential mechanism of drug resistance related to ER stress, emphasizing that ARHGEF2/EDN1 is a potential antiangiogenic therapeutic target in HCC. Lenvatinib’s flaw in ER stress-related apoptosis resistance may be addressed by an ARHGEF2 inhibitor.

## Supplementary information

Figure S1

Figure S2

Figure S3

Figure S4

Supplementary Figure legends

Table S1

Table S2

Table S3

Title Page

Original Data File

aj-checklist

## Data Availability

All data generated and analyzed during this study are included in this published article and supplementary information file. Materials generated in this study can be available upon reasonable request. The GEO accession number for mRNA-sequencing data of ARHGEF2 knockdown was GSE205757. The following GSE datasets are also used in this article, GSE36376, GSE10143 and GSE45267.
