# Supplementary figures and images for: ARHGEF2/EDN1 pathway participates in ER stress-related drug resistance of hepatocellular carcinoma by promoting angiogenesis and malignant proliferation
Source: Cell Death Dis. 2022 Jul 27;13(7):652. doi: 10.1038/s41419-022-05099-8 (PMC9329363; doi:10.1038/s41419-022-05099-8)

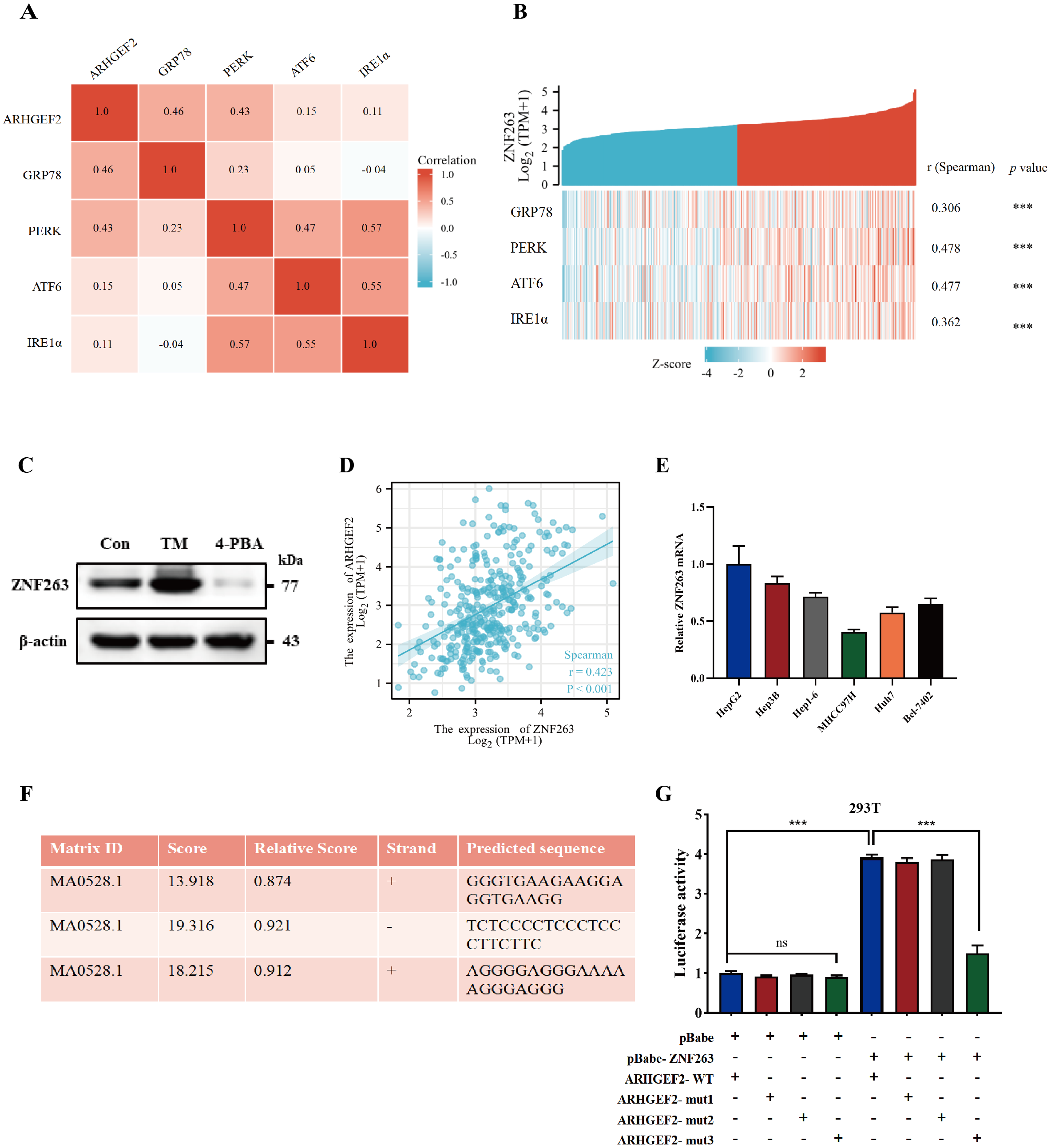

Supplement: Supplementary file 1 — Figure S1 [file 41419_2022_5099_MOESM1_ESM.png]

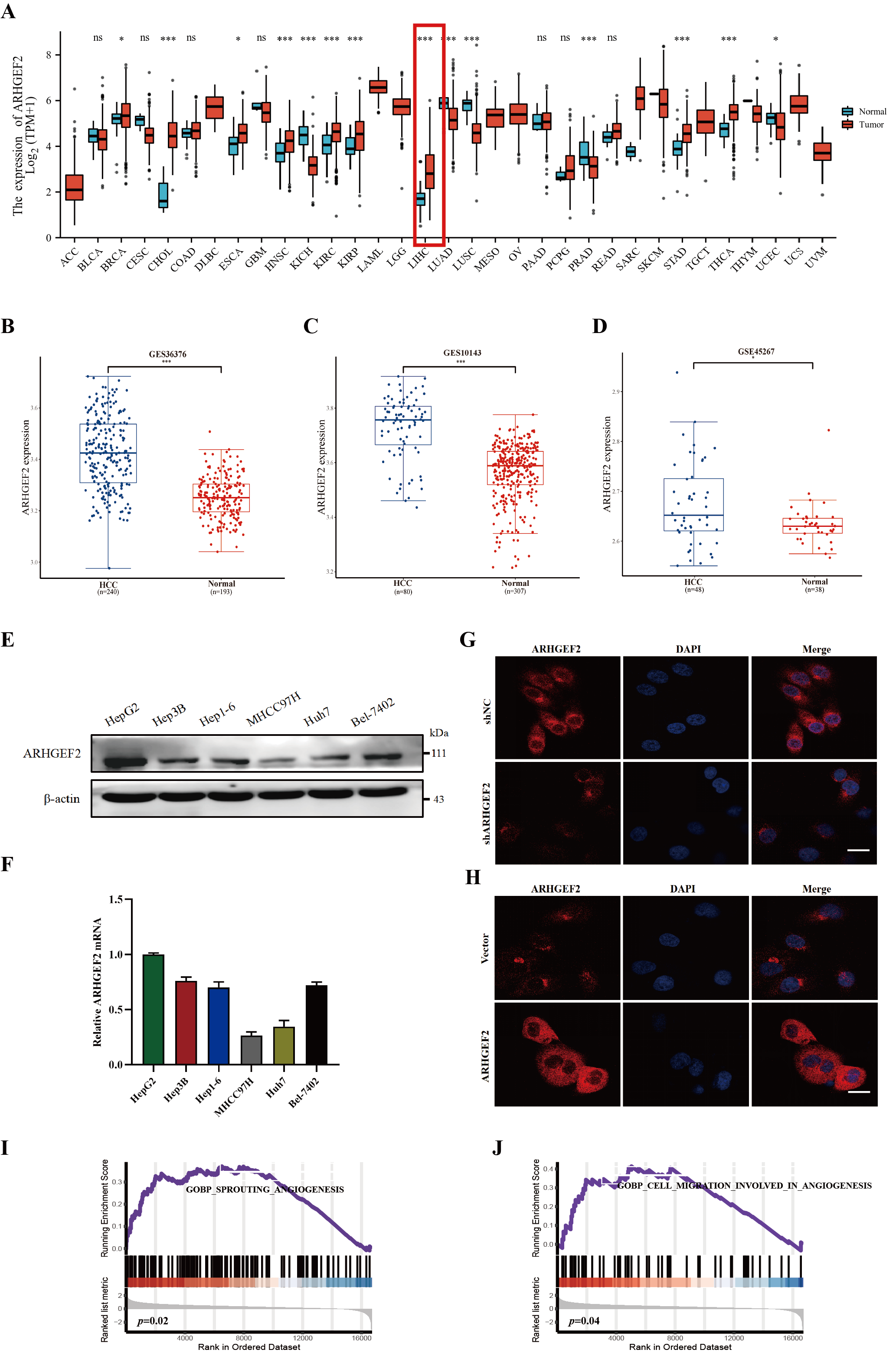

Supplement: Supplementary file 2 — Figure S2 [file 41419_2022_5099_MOESM2_ESM.png]

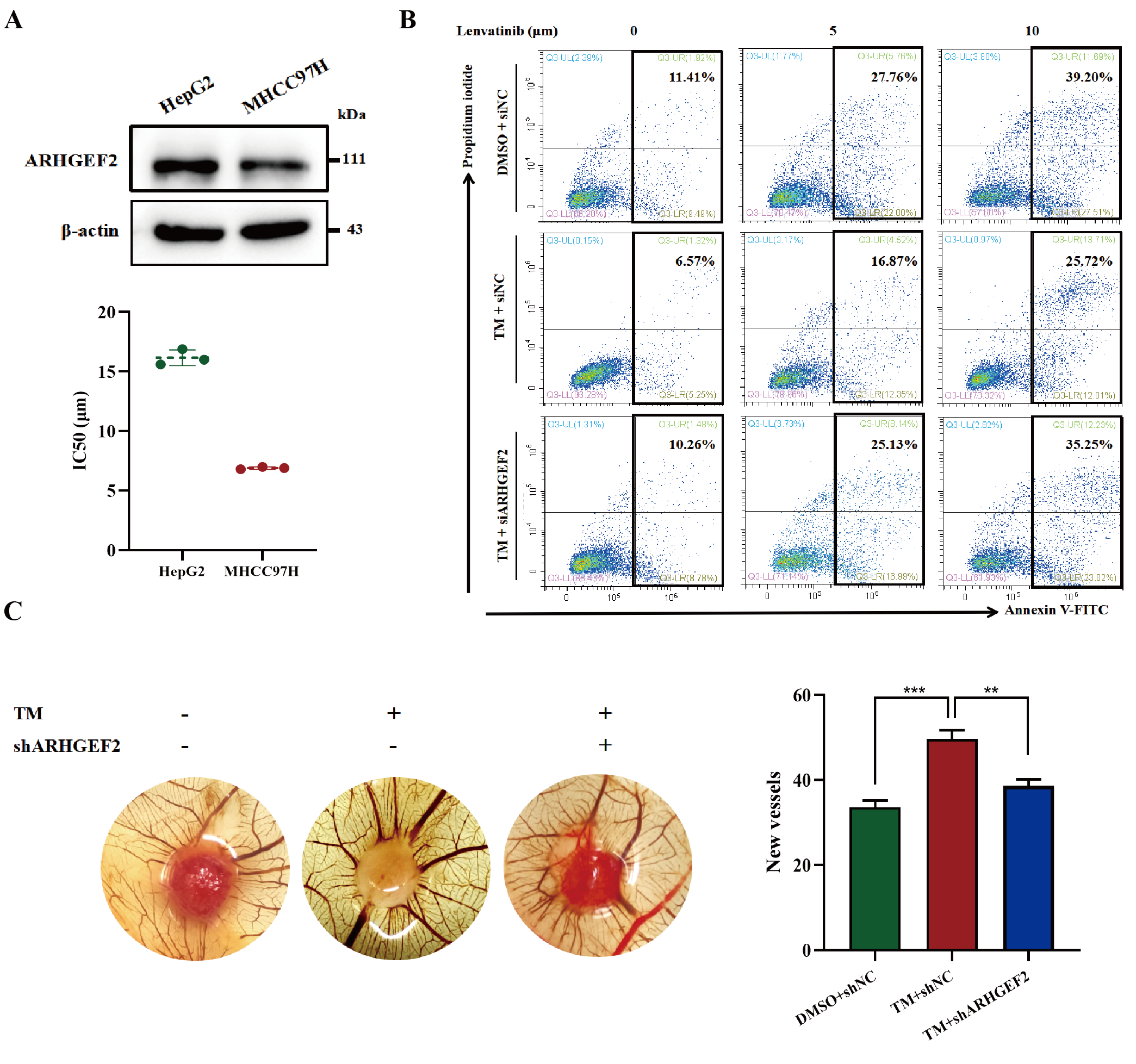

Supplement: Supplementary file 3 — Figure S3 [file 41419_2022_5099_MOESM3_ESM.png]

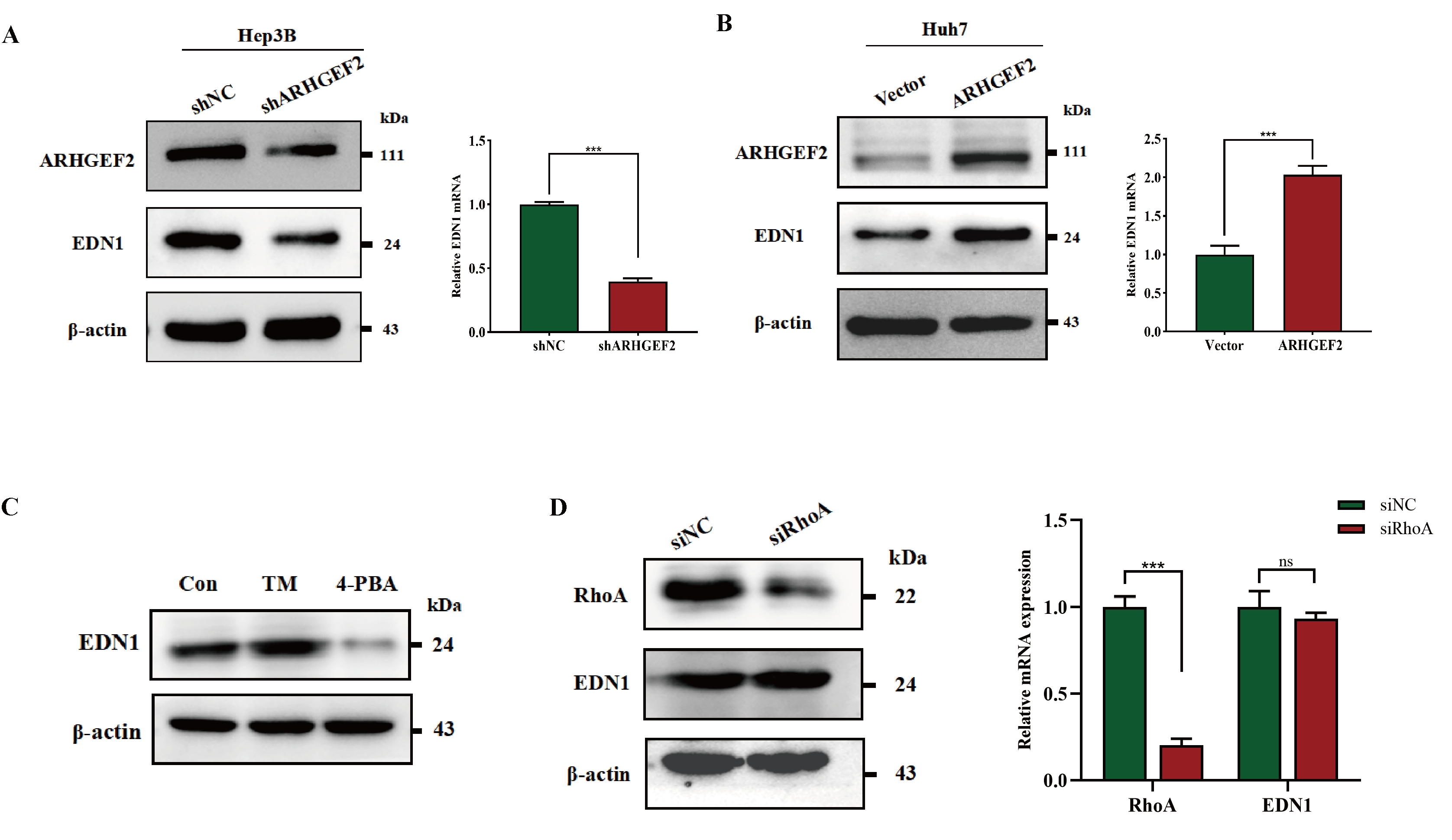

Supplement: Supplementary file 4 — Figure S4 [file 41419_2022_5099_MOESM4_ESM.png]
