# Supplementary material for: ARHGEF2/EDN1 pathway participates in ER stress-related drug resistance of hepatocellular carcinoma by promoting angiogenesis and malignant proliferation
Source: Cell Death Dis. 2022 Jul 27;13(7):652. doi: 10.1038/s41419-022-05099-8 (PMC9329363; doi:10.1038/s41419-022-05099-8)
Supplement: Supplementary file 5 — Supplementary Figure legends [file 41419_2022_5099_MOESM5_ESM.docx]

**Supplementary Figure 1.** (A) The correlation between ARHGEF2 and GRP78, PERK, ATF6, IRE1α were analyzed through the mRNA expression of 424 cases with HCC in TCGA database. (B)The correlation between ZNF263 and GRP78, PERK, ATF6, IRE1α were analyzed (TCGA). (C) The protein expression of ZNF263 was detected after HepG2 cells were treated with TM and 4-PBA. (D) The correlation between ZNF263 and ARHGEF2 in HCC according to mRNA expression in TCGA database. (E) qRT-PCR were used to detected the mRNA expression of ZNF263 in different hepatoma cell lines. (F) Three transcription sites were predicted using Jsapar database. (G) Dual-luciferase reporter assay was performed in 293T cells.

**Supplementary Figure 2.** (A) The expression of ARHGEF2 in 33 different cancer tissues and adjcent tissues were analyzed through the mRNA expression of 11093 cases in TCGA database. (B-D) Expression analysis of ARHGEF2 in HCC and normal tissues using GSE36376, GSE10143 and GSE45267. (E) The protein expression of ARHGEF2 in different hepatoma cell lines was detected by western blot. (F) qRT-PCR were used to detected the mRNA expression of ARHGEF2 in different hepatoma cell lines. (G) ARHGEF2 expression levels were confirmed by Immunofluorescence assay after ARHGEF2 knockdown in the HepG2 cell line. (H) ARHGEF2 expression levels were detected by Immunofluorescence assay after ARHGEF2 overexpression in the MHCC97H cell line. (I-J) Using 272 differentialy expression genes run GESA, demonstrating a significant correlation between the ARHGEF2 and sprouting angiogenesis (I), cell migration involved in sprouting angiogenesis (J).

**Supplementary Figure 3.**  (A) IC50 value of lenvatinib and the protein expression of ARHGEF2 in the HepG2 and MHCC97H cell lines. (B) The sensitivity of HepG2 cells to lenvatinib with different treatments were detected by Flow cytometry. (C) Representative images of CAM blood vessels stimulated with cell-conditioned media from the indicated cells.

**Supplementary Figure 4.** (A) The effect of ARHGEF2 knockdown on EDN1 levels were examined in Hep3B cells. (B) EDN1 protein and mRNA levels after ARHGEF2 overexpression were detected in Huh7 cells. (C) The protein expression of EDN1 was detected after HepG2 cells were treated with TM and 4-PBA. (D) Protein and mRNA levels of EDN1 were detected by Western blot and qRT-PCR after knockdown RhoA.
