# Supplementary material for: ARHGEF2/EDN1 pathway participates in ER stress-related drug resistance of hepatocellular carcinoma by promoting angiogenesis and malignant proliferation
Source: Cell Death Dis. 2022 Jul 27;13(7):652. doi: 10.1038/s41419-022-05099-8 (PMC9329363; doi:10.1038/s41419-022-05099-8)
Supplement: Supplementary file 6 — Table S1 [file 41419_2022_5099_MOESM6_ESM.docx]

Table S1. Antibody list

| Antibody | Concentration | | Company | Catalog Number |
| --- | --- | --- | --- | --- |
|  | Western blot | IHC |  |  |
| ARHGEF2 | 1:1000 | 1:500 | Abcam | ab155785 |
| β-actin | 1:1000 | N/A | Affinity | T0022 |
| EDN1 | 1:500 | N/A | Affinity | DF6125 |
| GRP78 | 1:1000 | N/A | Abcam | ab21685 |
| RhoA | 1:500 | N/A | Abcam | ab187027 |
| ZNF263 | 1:500 | 1:300 | Atlas Antibodies | HPA035086 |
| CD31 | N/A | 1:200 | Affinity | AF6191 |
| CD34 | N/A | 1:500 | Affinity | BF0423 |
| Ki67 | N/A | 1:200 | Affinity | [AF0198](http://www.affbiotech.cn/goods-897-AF0198-Ki67_Antibody.html) |

N/A: Not applicable
