# Supplementary material for: ARHGEF2/EDN1 pathway participates in ER stress-related drug resistance of hepatocellular carcinoma by promoting angiogenesis and malignant proliferation
Source: Cell Death Dis. 2022 Jul 27;13(7):652. doi: 10.1038/s41419-022-05099-8 (PMC9329363; doi:10.1038/s41419-022-05099-8)
Supplement: Supplementary file 7 — Table S2 [file 41419_2022_5099_MOESM7_ESM.docx]

Table S2. Primer sequences list

| Gene | Primer sequences | |
| --- | --- | --- |
|  | Forward (5’-3’) | Reverse (5’-3’) |
| ARHGEF2 | TCCCTCATTGACGAAGCAGA | GGTGCAGCTCTGTCTGGATT |
| CREB5 | CCCTGCCCAACCCTACAATG | GGACCTTGCATCCCCATGAT |
| GRP78 | CTGTCCAGGCTGGTGTGCTCT | CTTGGTAGGCACCACTGTGTTC |
| TRIB3 | TGCCCTACAGGCACTGAGTA | GTCCGAGTGAAAAAGGCGTA |
| HKDC1 | ATCCTGGCAAGCAGAGATACG | GACGCTCTGAAATCTGCCCT |
| NMNAT2 | GAGGCAGATATGGAGGTGATTG | TTTTTTATTTGCGGAGTATTGAGG |
| EDN1 | CAAGCAGGAAAAGAACTCAG | CTGGTTTGTCTTAGGTGTTC |
| ZNF263 | CTTGCGCTTCAGACGGTTC | CCCAAGCTCTCTGCATATCC |
| RhoA | CATCCGGAAGAAACTGGT | TCCCACAAAGCCAACTC |
| β-actin | CTGGCACCACACCTTCTACAATG | GGCGTACAGGGATAGCACAGC |
