# Supplementary material for: ARHGEF2/EDN1 pathway participates in ER stress-related drug resistance of hepatocellular carcinoma by promoting angiogenesis and malignant proliferation
Source: Cell Death Dis. 2022 Jul 27;13(7):652. doi: 10.1038/s41419-022-05099-8 (PMC9329363; doi:10.1038/s41419-022-05099-8)
Supplement: Supplementary file 8 — Table S3 [file 41419_2022_5099_MOESM8_ESM.docx]

Table S3. Sequences of siRNA

| Gene | Sequences | |
| --- | --- | --- |
|  | Sense (5’-3’) | Antisense (5’-3’) |
| ARHGEF2 | GCCCUGUACUUGAGUUUCATT | UGAAACUCAAGUACAGGGCTT |
| EDN1 | GGGCUGAAGACAUUAUGGATT | UCCAUAAUGUCUUCAGCCCTT |
| RhoA | GGCAGAGAUAUGGCAAACATT | UGUUUGCCAUAUCUCUGCCTT |
| ZNF263 | CCGUAUAAAUGUACCCUUUTT | AAAGGGUACAUUUAUACGGTT |
| Negative control | UUCUCCGAACGUGUCACGUTT | ACGUGACACGUUCGGAGAATT |
