# Supplementary material for: ARHGEF2/EDN1 pathway participates in ER stress-related drug resistance of hepatocellular carcinoma by promoting angiogenesis and malignant proliferation
Source: Cell Death Dis. 2022 Jul 27;13(7):652. doi: 10.1038/s41419-022-05099-8 (PMC9329363; doi:10.1038/s41419-022-05099-8)
Supplement: Supplementary file 9 — Title Page [file 41419_2022_5099_MOESM9_ESM.docx]

**ARHGEF2/EDN1 pathway participates in ER stress-related drug resistance of hepatocellular carcinoma by promoting angiogenesis and malignant proliferation**

**Running title:** ARHGEF2 promotes malignant progress in hepatocellular carcinoma Yue Zhu^1*^, Weiwei Liu^2*^, Zishu Wang^3*^, Yanfei Wang^1, 4^, Chaisheng Tan^1^, Zhipeng Pan^1^, Anqi Wang^1^, Jiatao Liu^5^, Guoping Sun^1^

^1^Department of Oncology, The First Affiliated Hospital of Anhui Medical University, Hefei, Anhui, China

^2^Department of Otorhinolaryngology, Head and Neck Surgery, The First Affiliated Hospital of Anhui Medical University, Hefei, Anhui, China

^3^Department of Oncology, The First Affiliated Hospital of Bengbu Medical College, Bengbu, Anhui, China

^4^Department of Integrated Traditional Chinese and Western Medicine, Anhui Medical University, Hefei, Anhui, China

^5^Department of Pharmacy, The First Affiliated Hospital of Anhui Medical University, Hefei, Anhui, China

^*^These authors contributed equally to this work.

**Corresponding author:** Guoping Sun, M.D., Ph.D., Department of Medical Oncology, The First Affiliated Hospital of Anhui Medical University, Hefei, Anhui, China, 230022. Tel.: +86-138-0560-9309. E-mail: [sungp@ahmu.edu.cn](mailto:sunguoping@ahmu.edu.cn)
