# Supplementary material for: ARHGEF2/EDN1 pathway participates in ER stress-related drug resistance of hepatocellular carcinoma by promoting angiogenesis and malignant proliferation
Source: Cell Death Dis. 2022 Jul 27;13(7):652. doi: 10.1038/s41419-022-05099-8 (PMC9329363; doi:10.1038/s41419-022-05099-8)
Supplement: Supplementary file 10 — Original Data File [file 41419_2022_5099_MOESM10_ESM.pdf]

Fig 1G Raw image

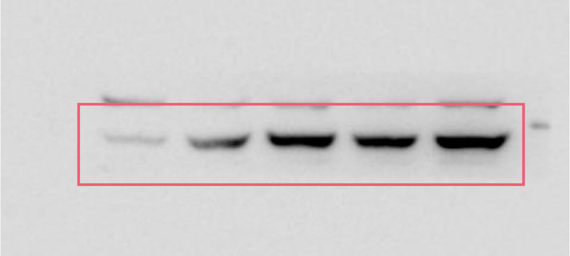

ARHGEF2

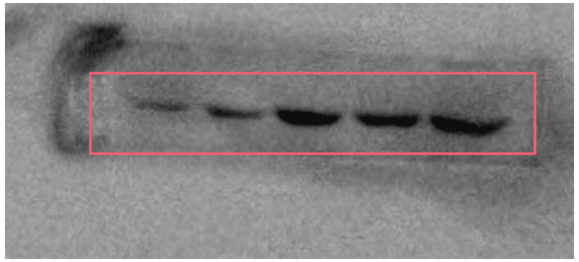

GRP78

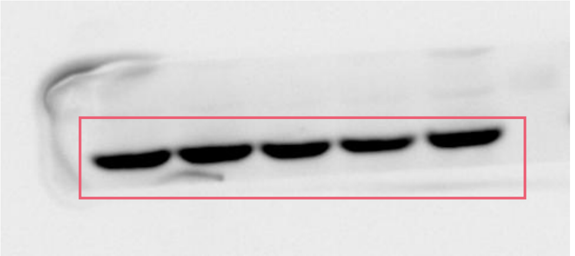

β-actin

Fig 1I Raw image

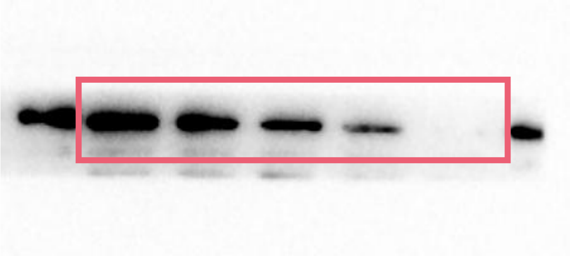

ARHGEF2

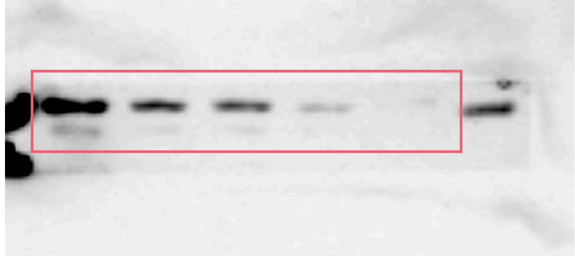

GRP78

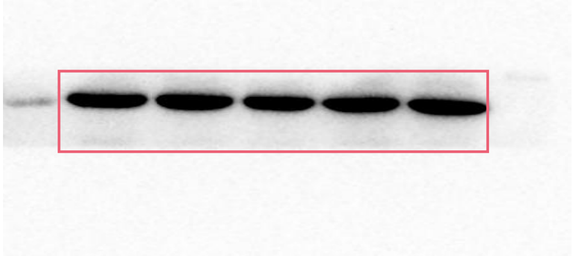

β-actin

Fig 2A-B Raw image

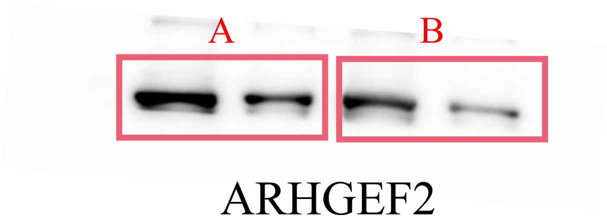

ARHGEF2

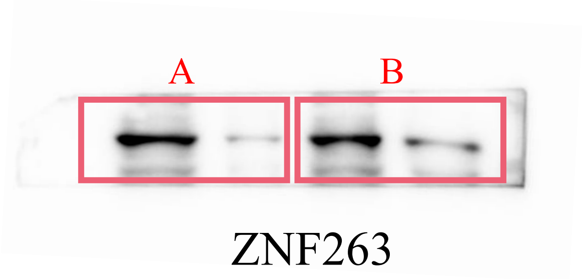

ZNF263

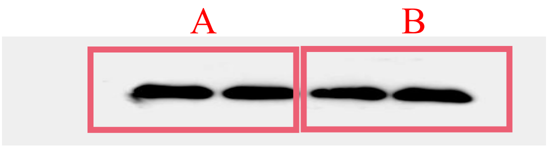

β-actin

Fig 2C-D Raw image

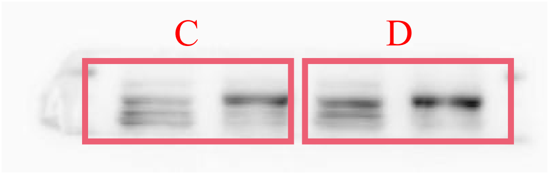

ARHGEF2

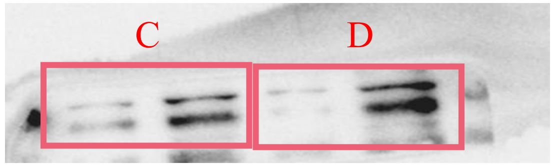

ZNF263

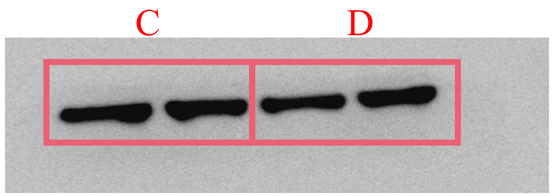

β-actin

Fig 2I Raw image

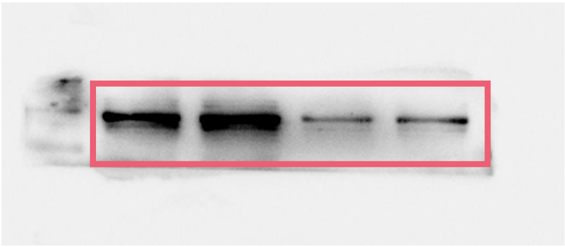

ARHGEF2

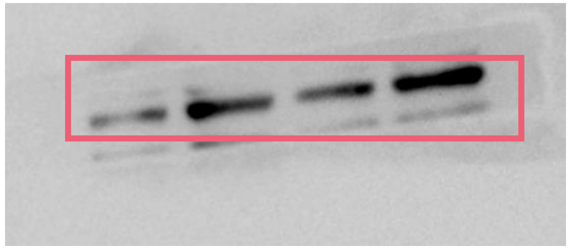

GRP78

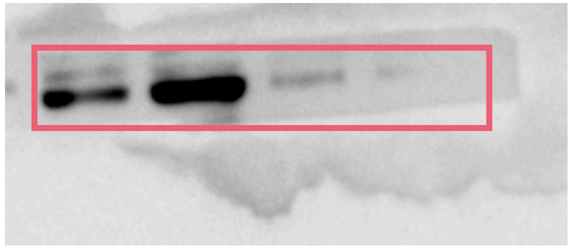

ZNF263

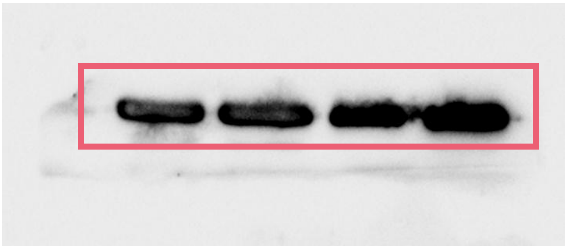

β-actin

Fig 3L Raw image

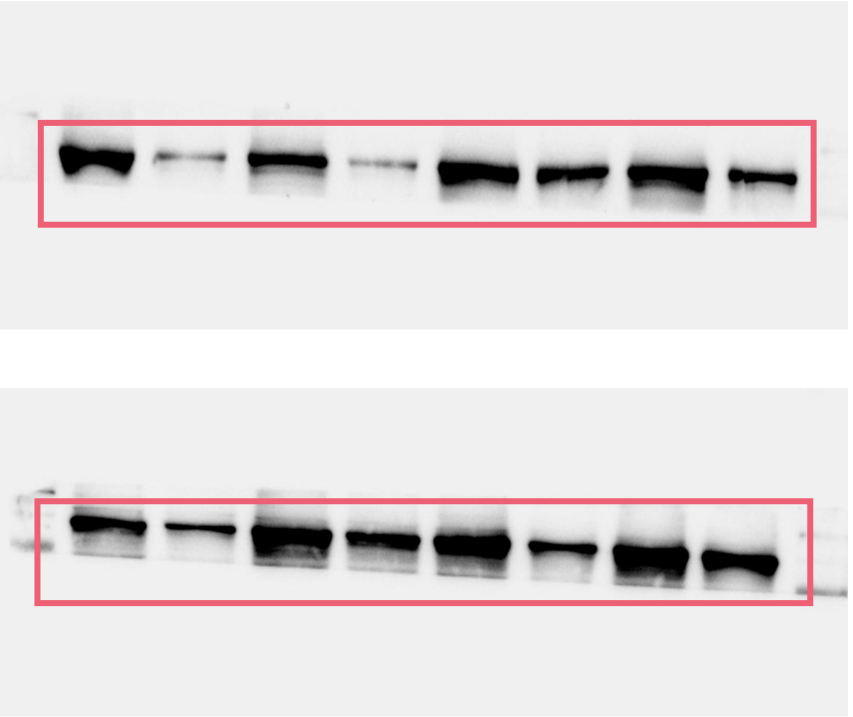

ARHGEF2

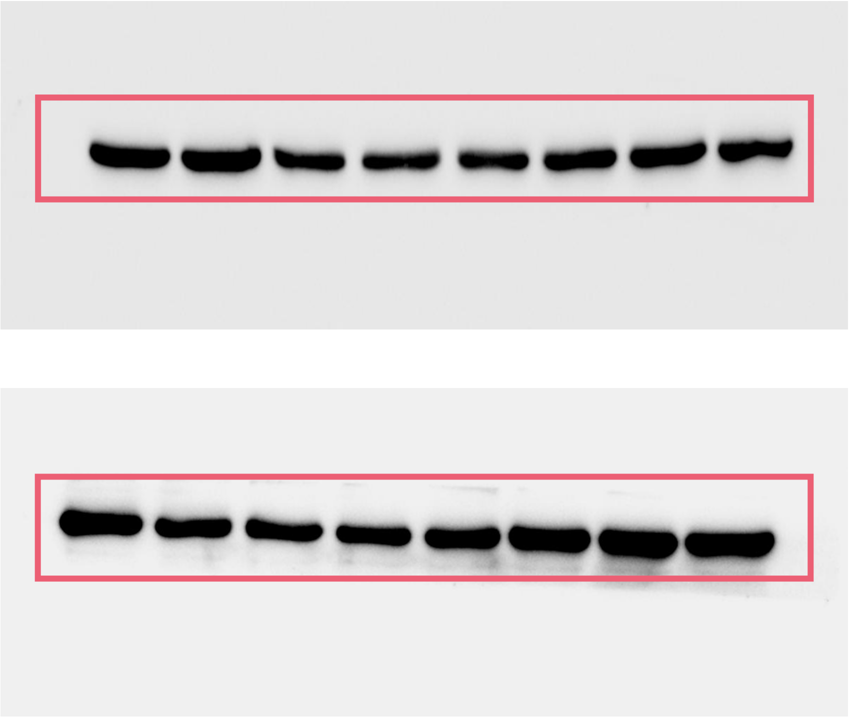

β-actin

Fig 4A, 4I Raw image

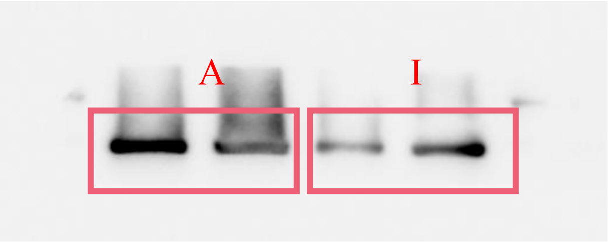

ARHGEF2

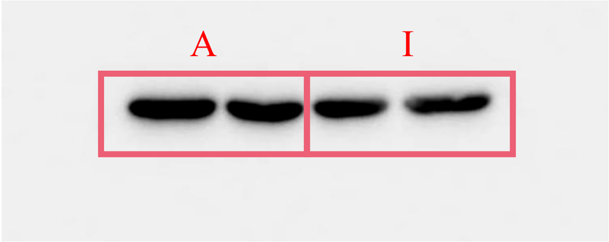

β-actin

Fig 7F, Fig S4A Raw image

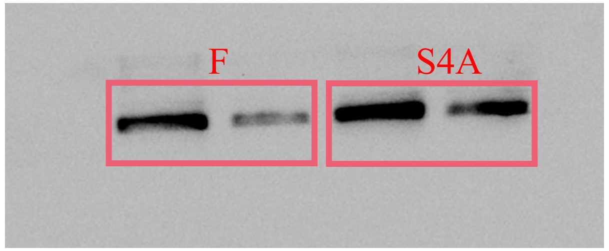

ARHGEF2

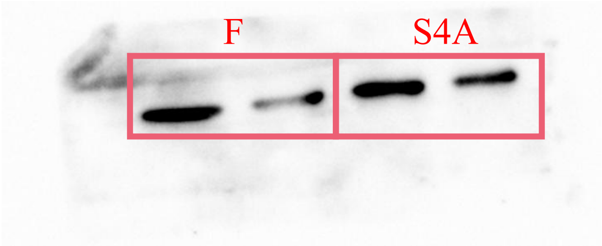

EDN1

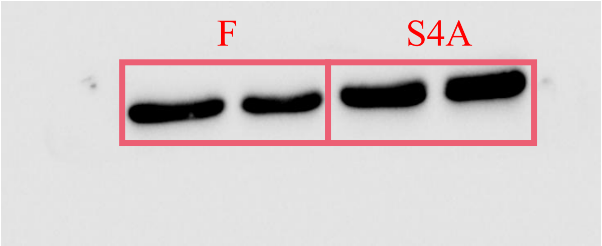

β-actin

Fig 7G, Fig S4B Raw image

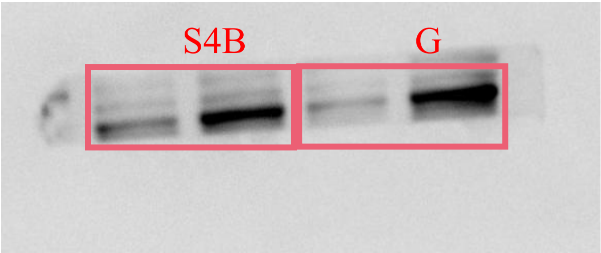

ARHGEF2

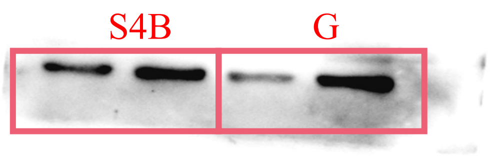

EDN1

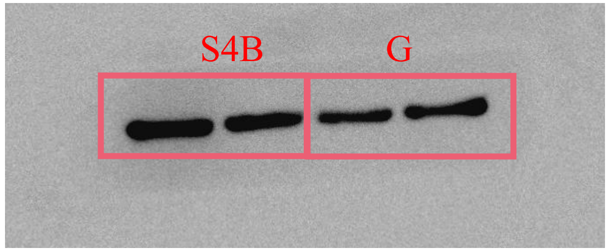

β-actin

Fig 7H Raw image

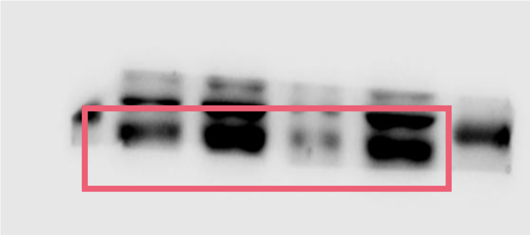

ZNF263

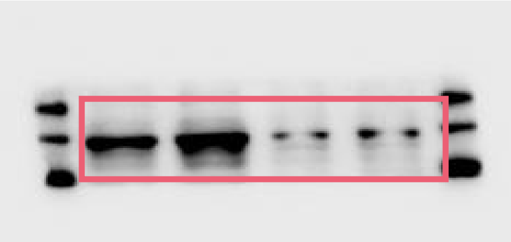

ARHGEF2

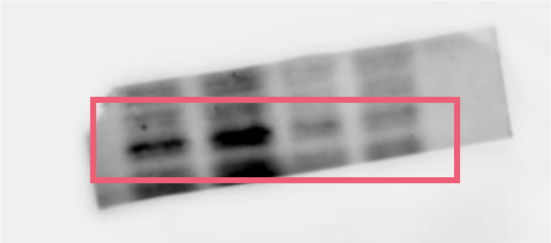

EDN1

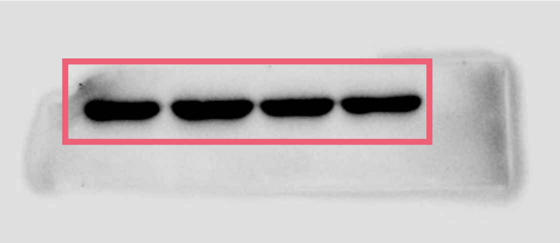

β-actin

Fig 7I Raw image

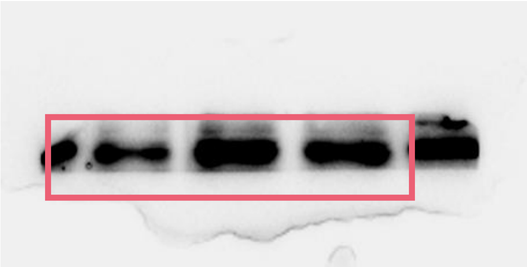

ZNF263

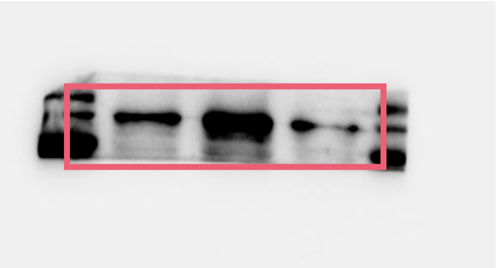

ARHGEF2

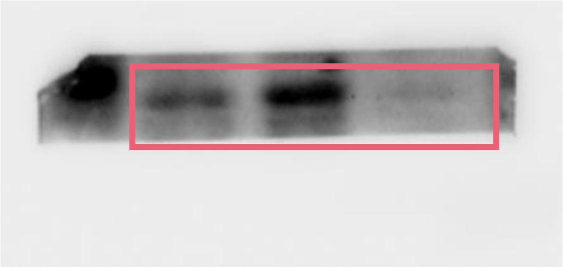

EDN1

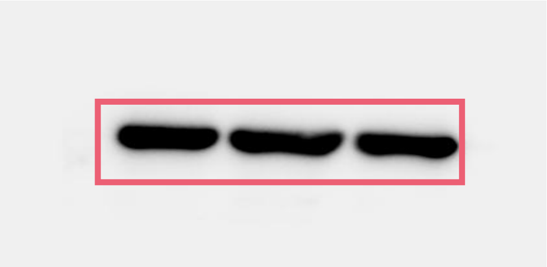

β-actin

Fig S1C Raw image

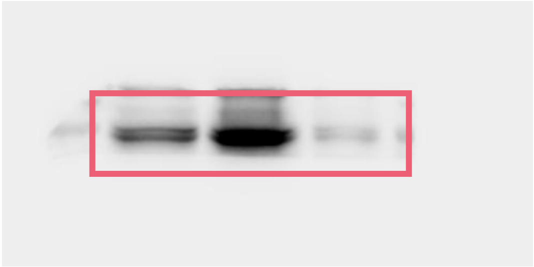

ZNF263

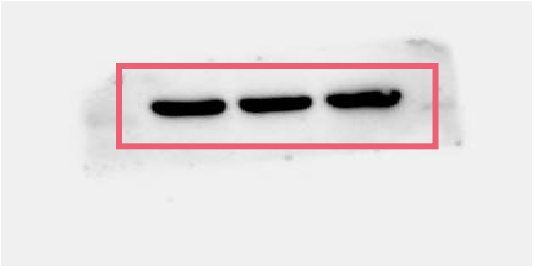

β-actin

Fig S2E Raw image

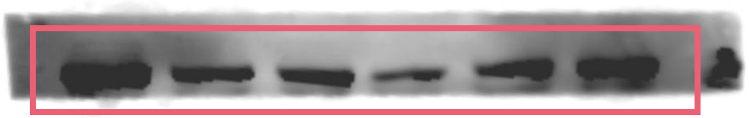

ARHGEF2

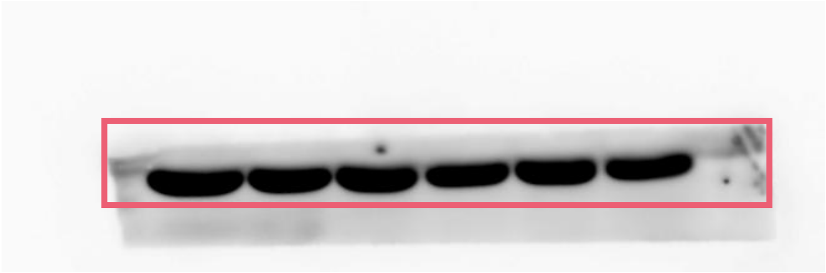

β-actin

Fig S3A Raw image

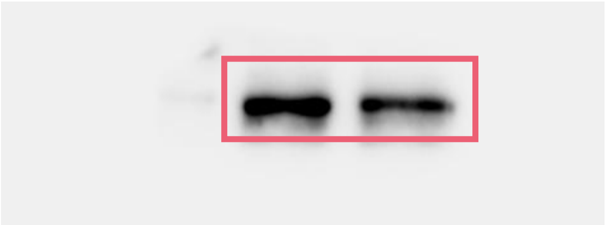

ARHGEF2

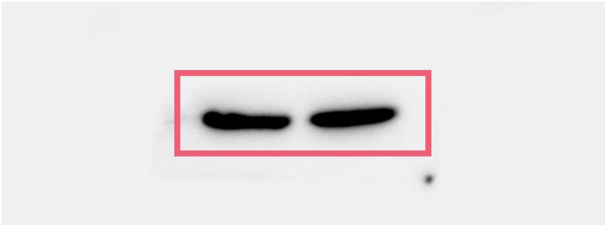

β-actin

Fig S4C Raw image

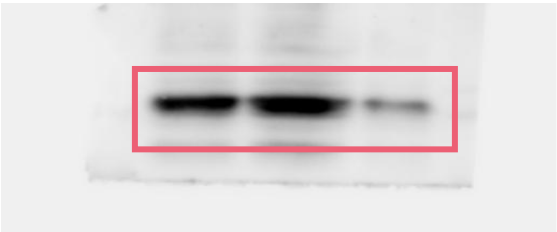

EDN1

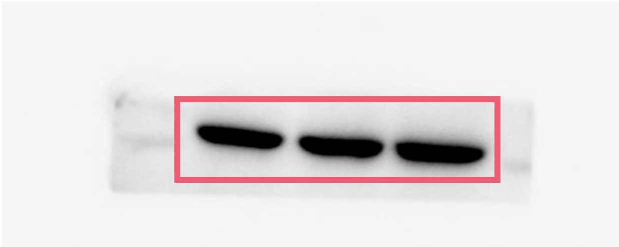

β-actin

Fig S4D Raw imag

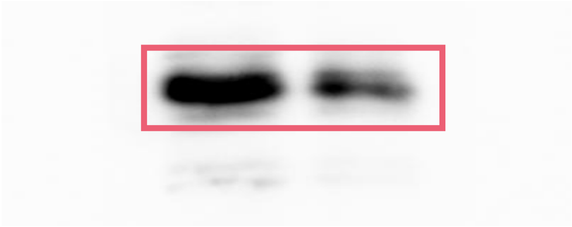

RhoA

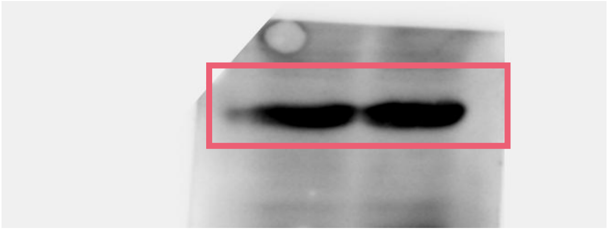

EDN1

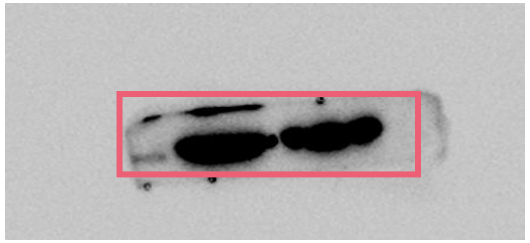

β-actin
